# New biologic (Ab-IPL-IL-17) for IL-17-mediated diseases: identification of the bioactive sequence (nIL-17) for IL-17A/F function

**DOI:** 10.1136/ard-2023-224479

**Published:** 2023-08-14

**Authors:** Anella Saviano, Adel Abo Manosour, Federica Raucci, Francesco Merlino, Noemi Marigliano, Anna Schettino, Mussarat Wahid, Jenefa Begum, Andrew Filer, Julia E Manning, Gian Marco Casillo, Marialuisa Piccolo, Maria Grazia Ferraro, Simona Marzano, Pasquale Russomanno, Rosa Bellavita, Carlo Irace, Jussara Amato, Mohammed Alfaifi, Peter Rimmer, Tariq Iqbal, Stefano Pieretti, Valentina Vellecco, Francesco Caso, Luisa Costa, Roberto Giacomelli, Raffaele Scarpa, Giuseppe Cirino, Mariarosaria Bucci, Helen M McGettrick, Paolo Grieco, Asif Jilani Iqbal, Francesco Maione

**Affiliations:** 1 ImmunoPharmaLab, Department of Pharmacy, School of Medicine and Surgery, University of Naples Federico II, Napoli, Italy; 2 Department of Clinical Laboratory Sciences, College of Applied Medical Sciences, King Khalid University, Abha, Saudi Arabia; 3 Department of Pharmacy, University of Naples Federico II, Napoli, Italy; 4 Institute of Inflammation and Ageing, College of Medical and Dental Sciences, University of Birmingham, Birmingham, UK; 5 Institute of Cardiovascular Sciences, College of Medical and Dental Sciences, University of Birmingham, Birmingham, UK; 6 BioChemLab, Department of Pharmacy, University of Naples Federico II, Napoli, Italy; 7 Department of Gastroenterology, Queen Elizabeth Hospital Birmingham, Birmingham, UK; 8 Institute of Microbiology and Infection, College of Medical and Dental Sciences, University of Birmingham, Birmingham, UK; 9 Department of Drug Research and Evaluation, Istituto Superiore di Sanità, Roma, Italy; 10 Department of Clinical Medicine and Surgery, University of Naples Federico II, Napoli, Italy; 11 Fondazione Policlinico Universitario, and Research Unit of Immuno-Rheumatology, Department of Medicine and Surgery, Campus Bio-Medico University, Via Alvaro del Portillo, 200, 00128 Roma, Italy, and Università Campus Bio-Medico di Roma, Via Alvaro del Portillo, 21, 00128 Roma, Italy, Roma, Italy; 12 Department of Cardiovascular Sciences, College of Medical and Dental Sciences, University of Birmingham, Birmingham, UK

**Keywords:** autoimmune diseases, inflammation, arthritis, rheumatoid, autoimmunity

## Abstract

**Objectives:**

Interleukin (IL) 17s cytokines are key drivers of inflammation that are functionally dysregulated in several human immune-mediated inflammatory diseases (IMIDs), such as rheumatoid arthritis (RA), psoriasis and inflammatory bowel disease (IBD). Targeting these cytokines has some therapeutic benefits, but issues associated with low therapeutic efficacy and immunogenicity for subgroups of patients or IMIDs reduce their clinical use. Therefore, there is an urgent need to improve the coverage and efficacy of antibodies targeting IL-17A and/or IL-17F and IL-17A/F heterodimer.

**Methods and results:**

Here, we initially identified a bioactive 20 amino acid IL-17A/F-derived peptide (nIL-17) that mimics the pro-inflammatory actions of the full-length proteins. Subsequently, we generated a novel anti-IL-17 neutralising monoclonal antibody (Ab-IPL-IL-17) capable of effectively reversing the pro-inflammatory, pro-migratory actions of both nIL-17 and IL-17A/F. Importantly, we demonstrated that Ab-IPL-IL-17 has less off-target effects than the current gold-standard biologic, secukinumab. Finally, we compared the therapeutic efficacy of Ab-IPL-IL-17 with reference anti-IL-17 antibodies in preclinical murine models and samples from patients with RA and IBD. We found that Ab-IPL-IL-17 could effectively reduce clinical signs of arthritis and neutralise elevated IL-17 levels in IBD patient serum.

**Conclusions:**

Collectively, our preclinical and in vitro clinical evidence indicates high efficacy and therapeutic potency of Ab-IPL-IL-17, supporting the rationale for large-scale clinical evaluation of Ab-IPL-IL-17 in patients with IMIDs.

**WHAT IS ALREADY KNOWN ON THIS TOPIC**

- Interleukin (IL) 17s cytokines (IL-17A, Il-17F and heterodimer IL-17A/F) are key drivers of inflammation that are functionally dysregulated in several human immune-mediated inflammatory diseases (IMIDs), such as rheumatoid arthritis (RA), psoriasis and inflammatory bowel disease (IBD).

**WHAT THIS STUDY ADDS**

- In this study, we identified for the first time the ‘essential’ amino acid sequence (nIL-17) responsible for IL-17A/F biological activity in both mouse and human. We have taken advantage of this knowledge to generate a novel antibody (Ab-IPL-IL-17) that specifically targets the active nIL-17 peptide sequence and has utility for understanding IL-17A/F biology/pathogenesis in mouse/human.

**HOW THIS STUDY MIGHT AFFECT RESEARCH, PRACTICE OR POLICY**

- We demonstrate that Ab-IPL-IL-17 is as effective as reference anti-IL-17 antibodies in reducing inflammatory processes, in preclinical models of IMIDs and in human clinical samples from IBD and RA. Importantly, Ab-IPL-IL-17 exhibited, in mice, significantly neutralising activity limiting inflammation and disease progression, with lower immunogenicity and adverse haematological side effects when compared with reference antibodies.

## Introduction

Evidence from basic research and clinical trials demonstrates that the interleukin (IL) 17 immune axis exerts distinct biological effects dependent on the tissue or disease context.^1^ IL-17-producing T cells (Th17) and innate immune cells (including neutrophils, monocytes and macrophages) play key protective roles in the immune response to various microbial pathogens.^2^ However, IL-17-driven responses are responsible for tissue damage linked to infection-associated immunopathology and can result in the development of immune-mediated inflammatory diseases (IMIDs),^3 4^ such as psoriasis, psoriatic arthritis (PsA), rheumatoid arthritis (RA), inflammatory bowel disease (IBD) and ankylosing spondylitis (AS).^5^ Dysregulation of IL-17A (and in a less extent, IL-17F and IL-17A/F) production and/or binding to its receptor(s) have been associated with IMID pathology,^6^ making this complex an attractive target for therapeutic interventions.^7^ Indeed, secukinumab, ixekizumab (anti-IL-17A antibodies) and bimekizumab (anti-IL-17A/F antibody) are already effective in treating plaque psoriasis, PsA and AS.^7^ Despite the potent blockade of cytokine signalling offered by these biological therapies, many patients have only partial or transient responses associated with various side effects. Therefore, identifying potential novel therapeutic targets or optimising those already available is urgently needed and will likely have a significant clinical benefit.^5 8^

IL-17 is composed of six family members, of which IL-17A and IL-17F are predominantly involved in driving inflammatory responses.^4^ Convincing evidence indicates that IL-17A/F use their C-terminal sequence to bind the heterodimeric receptor IL-17RA and IL-17RC.^9^ On binding, this receptor complex recruits the ubiquitin ligase Act-1 (via the SEF/IL-17R domain),^10^ which in turn recruits tumour necrosis factor α (TNF-α) receptor-associated factor 6 (TRAF6), leading to the activation of nuclear factor kappa B (NFκB) and the mitogen-activated protein kinase pathways. Activation of these pathways generates a plethora of inflammatory mediators, such as IL-1α/β, IL-6, IL-8 and TNF-α,^1 11 12^ which contribute to pathological processes in various IMIDs. Identification of the key active amino acids in the C-terminal sequence of IL-17A/F could, therefore, be critical in generating a more biologically active neutralising antibody with reduced off-target effects.^13^

Exploring murine and human IL-17A/F protein sequences, we have identified an essential 20-mer IL-17-derived peptide (nIL-17) that is responsible for the bioactivity of IL-17A, IL-17F and/or IL-17A/F heterodimer, mimicking a range of actions elicited by the full-length cytokines. Specifically, we demonstrate that nIL-17 activates IL-17RA/C-dependent intracellular signalling to induce activation of NIH-3T3 mouse embryonic fibroblast cells and human dermal blood endothelial cells (HDBECs) leading to increased cytokine, chemokine and adhesion molecules expression. Additionally, nIL-17 promoted leucocyte recruitment to pre-inflamed tissues in vivo (air pouch model) and in vitro (to inflamed endothelium). Subsequently, we developed a monoclonal neutralising antibody (Ab-IPL-IL-17) targeting nIL-17, which effectively reversed the actions of nIL-17 leading to reductions in chemokine, cytokine and adhesion molecule levels on target cells, as well as reducing the inflammatory infiltrate. Finally, we compared the therapeutic efficacy of Ab-IPL-IL-17 with reference anti-IL-17 antibodies in preclinical models of IMIDs, specifically arthritis and IBD. Crucially, Ab-IPL-IL-17 exhibited significantly more neutralising activity limiting inflammation and disease progression, with lower immunogenicity and adverse haematological side effects when compared with reference antibodies. Future studies and clinical trials will need to address the varying requirements of Ab-IPL-IL-17 as an alternative biological therapy for treating patients with IMIDs.

## Results

### Characterising the bioactive sequence in IL-17A and IL-17F

**Figure 1:**
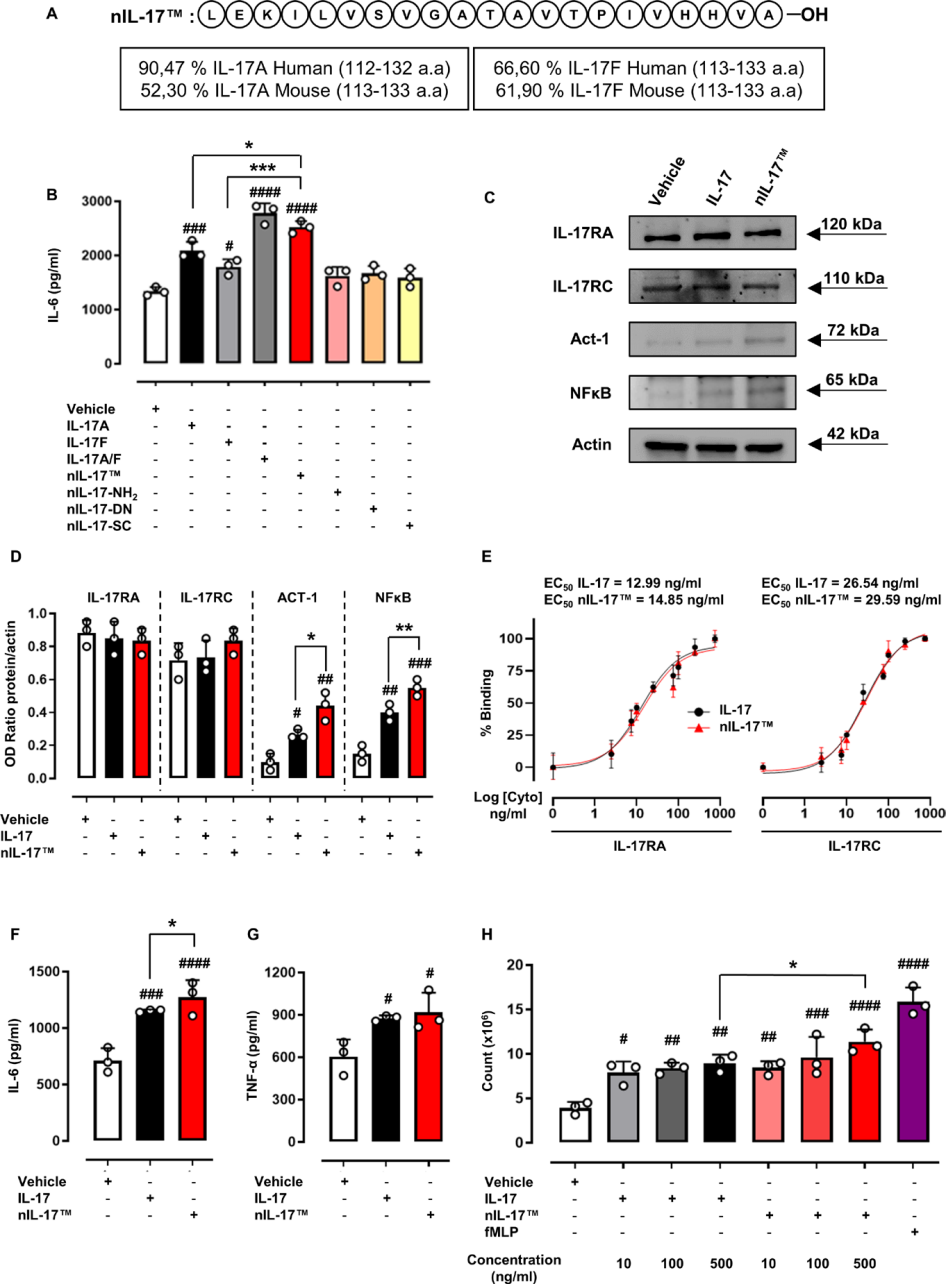
Biological characterisation of a novel IL-17-derived peptide (nIL-17). (A) Amino acids sequence of nIL-17 was obtained after a study of primary structures of both mouse/human IL-17A and IL-17F. (B) To assess the biological activity of nIL-17 peptide, IL-6 production was evaluated in NIH-3T3 cell supernatants following 24 hours of incubation in the presence of either IL-17A protein (50 ng/mL), IL-17F protein (50 ng/mL), IL-17A/F heterodimer (50 ng/mL), nIL-17 (50 ng/mL), nIL-17 (50 ng/mL) with terminal NH_2_ sequence, denatured (–DN) form or ‘scrambled’ (–SC) sequence (both at 50 ng/mL). (C)–(D) Whole cell lysates from NIH-3T3 cells stimulated with IL-17 or nIL-17 (50 ng/mL) were analysed, by western blot, for IL-17RA (~120 kDa), IL-17RC (~110 kDa), Act-1 (~72 kDa), NFκB (~65 kDa) and actin (~42 kDa) expression. Representative western blot images are shown from three pooled experiments with similar results. (E) To evaluate the binding interaction of nIL-17 with IL-17RA and IL-17RC, biotinylated IL-17 and nIL-17 (0–750 ng/mL) were co-incubated for 30 min with IL-17RA-Fc or IL-17RC-Fc prior to fluorescence being measured. (B)–(E) Data are presented as mean±SD of n=3 independent experiments. (F)–(G) Macrophages, derived from primary human CD14^+^ monocytes, were stimulated with LPS and IFN-γ (M1 stimuli) for 16 hours. Following differentiation, M1 macrophages were treated with IL-17 vehicle, IL-17 or nIL-17 (100 ng/mL) for 24 hours. Supernatants from all experimental conditions were assayed by ELISA for (F) IL-6 and (G) TNF-α. (H) Transwell chemotaxis assay was employed to determine the chemotactic activity of nIL-17. M199 media (final volume: 700 µL) was added to the bottom well of a Transwell-24 permeable support with 3.0 µm pores with IL-17 (10–500 ng/mL), nIL-17 (10–500 ng/mL) or fMLP (10^−6^ M as positive control). Neutrophils were added to the top chamber, which had a confluent stimulated (TNF-α and IFN-γ) HDBEC monolayer. (H) After 2 hours of incubation at 37°C, neutrophils were collected from the bottom of the wells and quantified using flow cytometry. (F)–(H) Data are presented as mean±SD of n=3 healthy donors. Statistical analysis was performed using the one-way analysis of variance test followed by Bonferroni. ^#^p≤0.05, ^##^p≤0.01, ^###^p≤0.001, ^####^p≤0.0001 vs vehicle group; *p≤0.05, **p≤0.01, ***p≤0.001 vs IL-17s group. fMLP, formyl-methionyl-leucyl-phenylalanine; HDBEC, human dermal blood endothelial cell; IFN-γ, interferon gamma; IL-17, interleukin 17; LPS, lipopolysaccharide; NFκB, nuclear factor kappa B; TNF-α, tumour necrosis factor α.

To identify the bioactive portion within IL-17A and IL-17F, we designed a series of peptides (online supplemental figure S1A–C), of different lengths, which mimic the C-terminal region of IL-17A/F, considered essential in the interaction with the receptor counterpart. These truncated peptides would meet the affinity/receptor interaction requirements of both murine and human IL-17A/F for their cognate receptors.^3 4^ Subsequently, we assessed the ability of these peptides to mimic the actions of native IL-17A, IL-17F and IL-17A/F heterodimer to induce IL-6 production from a murine embryonic fibroblast cell line NIH-3T3.^14^ Fibroblasts are a major cellular target for IL-17A, leading to the generation of several inflammatory cytokines (eg, IL-6 and IL-8), which drive the local inflammatory response.^9^ We found that only peptide 2 (named nIL-17) was able to promote IL-6 release to a greater extent when compared with both IL-17A and IL-17F at similar molar concentrations (from 0.610 nM to 0.725 nM) (figure 1A, online supplemental figure S1D). nIL-17A also displayed similar biological activity to the recombinant full-length native IL-17A/F heterodimer (figure 1A).

10.1136/ard-2023-224479.supp1 Supplementary data

Furthermore, our in vitro cytotoxic examination revealed a safe profile for nIL-17 in tested concentration on NIH-3T3 cell lines (online supplemental figure S2). Moreover, modifications through the replacement of carboxy (–CO_2_H) with amide (–CONH_2_) group at C-terminal (nIL-17A-NH_2_), removal of any tertiary structure by denaturing the peptide (nIL-17A-DN) or scrambling the amino acid sequence (nIL-17A-SC) had no effect on IL-6 production by NIH-3T3 (figure 1B). These data demonstrate for the first time that a 20-mer sequence from both murine and human IL-17A/F is responsible for IL-17s biological activity.

IL-17A, and to a lesser extent IL-17F and the heterodimer IL-17A/F, binding to the IL-17 receptor complex (IL-17RA/RC) leads to the recruitment of Act-1/TRAF6 and, ultimately, activation of inflammatory transcription factors via NFκB to induce gene expression.^15^ Indeed, we found that nIL-17 further amplified the expression of both Act-1 and NFκB, but not IL-17RA or IL-17RC, when compared with full-length native IL-17A protein (figure 1C,D; online supplemental figure S3). A similar observation was reported in mouse embryonic fibroblasts (NIH-3T3) and mouse macrophages (J774A.1) where both IL-17RA and IL-17RC expression remained unchanged following treatment with IL-17A.^14 16^ To confirm peptide–receptor interactions, biotinylated native IL-17A and IL-17F protein or nIL-17A were incubated with either mouse or human IL-17RA or IL-17RC and binding was assessed. nIL-17A displayed similar binding profiles to both receptors as was seen with full-length IL-17A/F (figure 1E; online supplemental figure S4, respectively).

**Figure 2:**
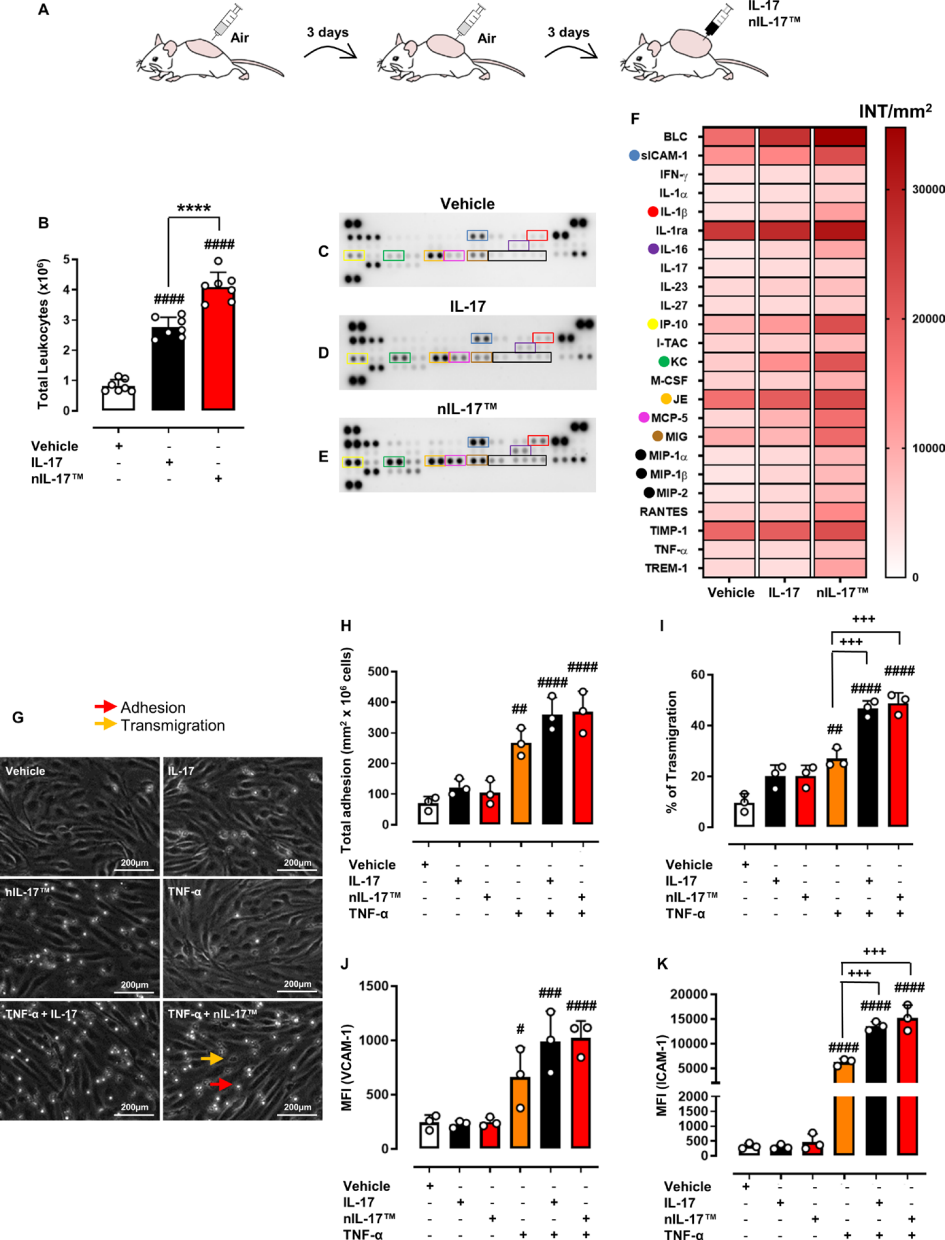
nIL-17 promotes leucocyte recruitment in vivo and migration in vitro. To evaluate the pro-inflammatory activity of nIL-17, we used a subchronic model of inflammation, the dorsal air pouch. (A) Mice were treated with IL-17 vehicle (0.5% CMC), IL-17 (1 µg/pouch) or nIL-17 (1 µg/pouch). (B) Total CD45^+^ leucocyte numbers were quantified by flow cytometry. (B) Data are presented as means±SD of n=7 mice per group. (C)–(E) Inflammatory supernatants obtained from pouch cavities were assayed using a Proteome Profiler Cytokine Array. (F) Densitometric analysis is presented as a heat map with dots indicating the most significant modulated cyto-chemokines mediators. (F) Data are presented as means±SD.D. of positive spots from three independent experiments run each with n=7 mice per group pooled. (G)–(I) To determine the impact of nIL-17 on leucocyte adhesion and transmigration on HDBEC, a static migration assay was used. HDBECs were treated with IL-17 vehicle (HCl 4 mM PBS), IL-17 (100 ng/mL) or nIL-17 (100 ng/mL), alone or in combination with TNF-α (100 U/mL) for 24 hours. (G) Representative images of the static adhesion assay are shown (200 µm magnification). PBMCs were added for 20 min on stimulated HDBEC, followed by washing to remove all non-adherent cells. Phase bright PBMCs were considered (H) adherent (red arrow), whereas phase-dark were quantified as (I) transmigrated (% of adherent cells) (orange arrow). (J)–(K) VCAM-1 and ICAM-1expression on HDBECs was quantified by flow cytometry. (H)–(K) Data are presented as means±SD of n=3 independent healthy donors. Statistical analysis was conducted by one or two-way analysis of variance test followed by Bonferroni’s correction for multiple comparisons. ^#^p≤0.05, ^##^p≤0.01, ^###^p≤0.001, ^####^p≤0.0001 vs vehicle group; ****p≤0.0001 vs IL-17 group; ^+++^p≤0.001 vs TNF-α group. CMC, carboxymethyl cellulose; HDBECs, human dermal blood endothelial cells; ICAM-1, intercellular adhesion molecule-1; IL-17, interleukin 17; PBMCs, peripheral blood mononuclear cells; PBS, phosphate-buffered saline; TNF-α, tumour necrosis factor α; VCAM-1, vascular cell adhesion molecule-1.

To gain a better understanding of how nIL-17 binds IL-17RA and IL-17RC receptors at an atomic level, molecular docking studies were performed. The 3D structure of nIL-17 peptide was predicted using the PEP-FOLD4 computational tool^17^ and experimentally analysed by circular dichroism (online supplemental figure S5-I-A). Using the BeStSel method,^18^ we selected a structural model of nIL-17 that consisted of two β-strands followed by a short α-helix (online supplemental figure S5-I-B; online supplemental auxiliary table 1). This model showed the closest structural similarity to the receptor-binding region of human IL-17A. The three-dimensional (3D) structures of IL-17RA and IL-17RC proteins were obtained from the crystal structure of the two receptors in complex with human IL-17A (protein data bank id: 7ZAN), also considering the similarity between IL-17RC isoform 1 used in our biological tests and isoform 2 present in the 3D structure (online supplemental auxiliary table 2). Subsequent docking analysis predicted that nIL-17 interacts with IL-17RA and IL-17RC in a manner similar to that of the C-terminal region of IL-17A, whose sequence it mimics (online supplemental figure S5-II). In particular, nIL-17 interacts with the IL-17RA binding pocket between two type III fibronectin domains, with D1 binding the N-terminal region of nIL-17 and D2 interacting mainly with the C-terminal α-helix (online supplemental figure S5-III). While the predicted binding of nIL-17 was mostly superimposable with IL-17A homodimer, there were significant differences in the positioning of nIL-17 N-terminal region and in the interaction with the receptor amino acids (online supplemental figure S5-III-B,C). The predicted binding mode of nIL-17 with IL-17RC was similar to that seen for IL-17RA, except no interactions between the N-terminal region of nIL-17 and the D1 domain were predicted (online supplemental figure S5-IV). The higher affinity of nIL-17 for IL-17RA compared with the other peptides and the IL-17A C-terminal region may be attributable to the multiple interactions formed with the D2 domain that anchors the peptide to the receptor. This binding mode may be possible due to the peculiar structural conformation of nIL-17, permitting closure of the binding site between the two type III fibronectin domains (online supplemental auxiliary figure 1), thereby improving the biological activity of nIL-17 compared with other peptides (online supplemental figure S1). Conversely, the limited interaction between nIL-17 and IL-17RC may in part be due to the lack of closure of the binding cavity (online supplemental auxiliary figure 2), which may explain the differential binding activity of nIL-17 toward the two receptors. Collectively, these data support our hypothesis that the bioactive region within both IL-17A and IL-17F meets the affinity/receptor interaction requirements of both murine and human cognate receptors.

**Table 1:** Haematological parameters of vehicle, Ab-IPL-IL-17, MAB421 and secukinumab-treated mice

	Vehicle (n=5)	Ab-IPL-IL-17 (n=5)	MAB421 (n=5)	Secukinumab (n=5)	Bimekizumab (n=5)
WBC† (×10^3^ /µL)					
2 hours	3.60±1.43	3.78±1.29	3.84±1.30	3.96±1.19	3.90±0.91
24 hours	3.52±0.91	4.06±1.70	4.08±1.22	4.44±1.88	4.60±1.70
72 hours	3.38±1.15	4.48±1.99	4.56±2.25	5.10±2.34	4.84±2.49
7 days	3.60±1.29	4.02±1.90	4.08±1.77	4.74±1.30	5.08±1.37
14 days	3.60±0.83	3.88±0.81	3.70±1.30	3.08±0.86	3.00±0.68
21 days	3.34±0.94	3.44±1.40	3.30±1.58	3.280±1.47	3.32±1.18
MID† (10^3^ /µL)					
2 hours	0.34±0.23	0.38±0.19	0.36±0.23	0.38±0.31	0.36±0.24
24 hours	0.34±0.21	0.48±0.19	0.46±0.13	0.44±0.25	0.42±0.31
72 hours	0.38±0.15	0.56±0.23	0.54±0.21	0.40±0.25	0.42±0.22
7 days	0.34±0.25	0.46±0.09	0.42±0.26	0.40±0.16	0.46±0.18
14 days	0.38±0.19	0.44±0.11	0.40±0.16	0.38±0.13	0.40±0.10
21 days	0.34±0.22	0.36±0.11	0.36±0.17	0.34±0.15	0.32±0.16
GRA† (10^3^ /µL)					
2 hours	0.20±0.16	0.22±0.19	0.20±0.16	0.22±0.16	0.24±0.11
24 hours	0.20±0.10	0.26±0.15	0.26±0.13	0.24±0.11	0.22±0.16
72 hours	0.22±0.11	0.38±0.08	0.34±0.11	0.30±0.16	0.30±0.10
7 days	0.20±0.10	0.28±0.08	0.28±0.13	0.24±0.13	0.26±0.11
14 days	0.18±0.08	0.26±0.11	0.24±0.13	0.20±0.10	0.20±0.12
21 days	0.20±0.10	0.24±0.05	0.24±0.11	0.20±0.10	0.22±0.13
RBC† (×10^6^ /µL)					
2 hours	5.99±1.29	5.74±1.22	5.56±1.12	5.57±1.18	5.84±1.62
24 hours	6.09±1.203	5.96±1.05	5.66±1.21	4.48±1.11	4.63±0.99
72 hours	6.12±1.02	6.30±0.78	6.29±1.19	3.7±1.05 *	3.79±1.02*
7 days	6.14±1.19	6.29±0.95	6.09±0.66	4.10±0.78	4.18±0.93
14 days	6.12±1.03	6.47±1.00	6.12±0.46	5.98±1.42	5.81±1.41
21 days	6.15±0.98	6.41±0.96	6.27±0.54	6.25±1.18	6.37±1.11
Hb† (g/dL)					
2 hours	9.84±2.22	10.58±1.55	10.10±1.87	9.92±1.78	9.88±1.02
24 hours	10.08±1.74	10.72±1.50	10.26±1.62	8.88±1.65	9.08±1.55
72 hours	10.20±1.73	10.84±1.56	10.26±1.71	6.92±1.53*	7.10±1.38*
7 days	10.32±1.366	10.74±1.03	10.20±1.17	7.76±1.31	8.10±1.33
14 days	10.28±1.593	10.96±1.23	10.34±1.30	9.480±1.36	9.06±1.84
21 days	10.22±1.897	10.90±1.06	10.42±1.08	10.16±1.52	10.06±1.27
HCT† (%)					
2 hours	30.24±6.13	29.84±4.21	28.62±3.68	29.56±2.26	29.96±2.29
24 hours	30.30±5.25	30.24±3.57	28.98±3.97	28.86±1.80	29.56±2.16
72 hours	31.00±5.00	31.64±3.64	30.28±4.38	21.30±5.47**	22.04±4.37**
7 days	30.58±4.76	31.46±2.97	29.58±4.05	22.94±4.68*	22.48±3.74*
14 days	30.70±5.35	31.86±1.87	30.48±1.80	27.88±2.63	28.36±3.19
21 days	31.00±4.61	31.88±2.12	31.16±2.23	29.04±2.30	29.20±2.70
MCV† (fL)					
2 hours	62.96±10.27	60.62±8.00	60.76±7.5	61.86±8.95	61.22±5.70
24 hours	64.16±10.23	62.32±8.25	61.22±6.33	63.60±8.78	62.32±10.33
72 hours	64.24±11.81	62.98±8.30	61.34±8.01	81.12±8.84	80.98±9.17
7 days	63.06±9.67	62.60±9.27	62.00±6.18	71.98±8.64	72.78±8.77
14 days	63.12±7.37	62.62±8.51	60.76±5.23	63.46±8.13	61.10±9.83
21 days	64.10±8.72	63.18±8.74	62.36±4.85	62.56±7.90	62.92±6.53
MCH† (pg)					
2 hours	16.88±1.83	17.48±1.32	17.52±1.06	17.58±1.42	16.98±1.38
24 hours	17.28±2.25	17.32±1.75	17.90±1.38	19.10±1.88	18.92±1.42
72 hours	17.40±2.02	17.70±1.44	17.56±1.12	21.78±4.20*	21.46±2.04*
7 days	17.42±1.72	17.54±1.19	17.60±0.62	21.14±4.82	21.22±3.74
14 days	17.76±1.77	17.74±1.18	17.44±0.70	17.50±1.09	17.48±1.38
21 days	17.04±1.66	17.08±1.18	17.40±0.95	17.62±1.05	17.96±1.57
MHCH† (g/dL)					
2 hours	30.48±3.57	29.94±3.12	30.76±4.19	30.60±4.27	30.00±3.06
24 hours	30.80±3.80	29.52±3.76	30.74±3.74	31.54±4.15	30.70±3.18
72 hours	31.14±3.94	30.66±3.54	30.28±4.05	30.90±6.72	30.62±5.57
7 days	30.64±3.74	29.99±2.86	30.50±5.29	29.50±5.03	29.36±5.05
14 days	30.82±3.00	30.72±2.61	30.34±4.24	29.64±4.38	29.96±4.45
21 days	30.46±4.04	30.18±3.55	30.00±4.85	30.34±4.54	30.50±4.52
RDW† (%)					
2 hours	17.94±2.17	18.08±2.84	18.06±2.47	18.14±1.87	18.82±1.75
24 hours	17.72±2.03	18.04±2.91	18.54±1.56	18.24±1.12	18.42±1.18
72 hours	18.08±2.61	16.90±2.08	16.96±1.55	16.86±3.47	17.12±2.05
7 days	18.30±2.72	17.94±1.94	17.88±1.33	18.38±1.90	18.14±3.00
14 days	18.06±3.03	17.86±1.76	17.76±1.90	18.06±2.06	18.26±2.28
21 days	18.40±2.50	17.88±1.94	17.60±0.58	18.34±1.33	18.02±1.34
MPV† (fL)					
2 hours	4.88±0.64	4.74±0.56	4.66±0.50	4.54±0.5771	4.64±0.86
24 hours	4.92±0.57	4.82±0.53	4.96±0.68	5.16±0.8764	5.00±1.12
72 hours	4.98±0.52	4.96±0.53	5.08±0.58	7.80±1.733****	7.96±1.95****
7 days	4.88±0.62	5.00±0.67	4.84±0.54	6.58±1.242*	6.78±1.09*
14 days	4.90±0.50	5.12±0.67	4.98±0.57	5.86±0.5128	5.66±0.92
21 days	5.04±0.55	5.12±0.40	4.92±0.73	4.92±0.4207	5.02±0.76
PCT† (%)					
2 hours	0.27±0.09	0.28±0.08	0.28±0.08	0.27±0.08	0.26±0.07
24 hours	0.28±0.08	0.28±0.07	0.28±0.07	0.25±0.09	0.26±0.06
72 hours	0.28±0.10	0.29±0.05	0.28±0.07	0.16±0.06	0.17±0.03
7 days	0.28±0.09	0.28±0.05	0.28±0.07	0.21±0.05	0.22±0.07
14 days	0.28±0.10	0.28±0.05	0.29±0.06	0.25±0.05	0.26±0.05
21 days	0.29±0.07	0.28±0.05	0.28±0.07	0.28±0.04	0.28±0.03
PWD† (%)					
2 hours	15.98±2.63	16.24±2.34	16.28±2.31	16.30±2.15	16.80±1.42
24 hours	15.86±2.77	16.58±2.61	16.36±2.37	16.06±2.30	16.04±1.30
72 hours	16.02±2.52	16.30±1.98	16.36±2.25	18.02±2.61	17.94±2.99
7 days	15.76±2.52	16.52±2.24	16.34±2.40	15.54±2.91	16.04±2.71
14 days	16.04±2.68	16.72±1.73	16.28±1.98	15.98±2.03	16.02±1.63
21 days	16.10±2.37	16.78±1.49	16.24±2.35	16.14±1.80	16.24±1.83

Serum samples collected by intracardiac puncture of vehicle, Ab-IPL-IL-17, MAB421, secukinumab or bimekizumab (100 µg/mouse) treated mice were assessed for haematological parameters (WBC, MID, GRA, RBC, Hb, HCT, MCV, MCH, MHCH, RDW, MPV, PCT and PWD) at indicated time-points. Results obtained were expressed as the mean±SD Statistical analysis was performed by using one-way analysis of variance test followed by Bonferroni’s for multiple comparisons. *p≤0.05, **p≤0.01, ****p≤0.0001 vs vehicle group (n=5 mice per group).

†Mean±SD.

GRA, granulocytes; Hb, haemoglobin; HCT, haematocrit; MCH, mean corpuscular haemoglobin; MCHC, mean corpuscular haemoglobin concentration; MCV, mean cell volume; MID, minimum inhibitory dilution; MPV, mean platelet volume; PCT, plateletcrit; PWD, platelet distribution width; RBC, red blood cells; RDW, red blood cells distribution width; WBC, white blood cells.

### nIL-17 is a potent activator of inflammatory response

IL-17A, and in part IL-17F, can modulate a variety of leucocyte functions, having a broad and wide-ranging impact on inflammatory responses.^19^ In the context of the myeloid lineage, it has previously been shown that treatment with IL-17A can amplify the production of inflammatory cytokines from human M1 macrophages.^20^ In agreement with this study, nIL-17 significantly increased IL-6 and TNF-α release from M1 macrophages to a similar degree as seen with native full-length IL-17A (figure 1F,G). In line with previous studies,^21^ this response was specific for M1 macrophages, and not seen in M0 or M2 macrophages (online supplemental figure S6A,B). Furthermore, there were no intrinsic differences in IL-17RA or IL-17RC expression following IL-17A or nIL-17 treatment on any of the macrophage subsets (online supplemental figure S6C–E). These data demonstrate that nIL-17 retains a similar inflammatory amplification activity as that observed with the native full-length protein.

In the context of inflammatory cell recruitment, IL-17A, but not IL-17F, can directly function as a chemotactic agent for neutrophils promoting their entry into inflamed tissues.^22^ Moreover, IL-17A acts synergistically with TNF-α to increase the expression of neutrophil capture receptors (E-selectin and P-selectin) and presentation of neutrophil specific chemokines (chemokine C–X–C motif ligand (CXCL) 1, 2, 8) by HDBEC.^23^ We, therefore, assessed the chemotactic potential of nIL-17 to drive neutrophil migration through inflamed endothelial cells (ECs) (figure 1H). Notably, we found that nIL-17 significantly increased neutrophil migration in a concentration-dependent manner, unlike native IL-17A (figure 1H). Interestingly, 500 ng/mL nIL-17 had a greater chemotactic capacity to drive neutrophil migration when compared with native IL-17A at the same concentration (figure 1H).

To validate these findings in vivo, we used the myeloid-driven subchronic model of inflammation, the mouse dorsal air pouch (figure 2A), where we previously demonstrated that IL-17A preferentially increases the recruitment of pro-inflammatory Ly6C^hi^ monocytes and Gr1^+^ neutrophils in acute and chronic inflammatory settings.^16 24 25^ Native murine IL-17A or nIL-17 were administered on day 6 following establishment of the air pouch. We observed a significant increase in CD45^+^ leucocytes recruited in response to IL-17A, which was further exacerbated (~48%) in the presence of nIL-17 (figure 2B). Moreover, in the presence of a commercially available neutralising IL-17A monoclonal antibody (MAB421), this effect was lost (online supplemental figure S7). Such effects have been previously described in models of endotoxin-induced lung inflammation.^26^ Interestingly, blocking the neutrophil (IL-8/keratinocyte-derived cytokine (KC)) or monocyte (JE/monocyte chemoattractant protein-1 (MCP-1)) chemokines simultaneously with administrating nIL-17 significantly impaired leucocyte recruitment to the air pouch (online supplemental figure S7), indicating that nIL-17 drives neutrophil and monocyte infiltration via indirect release of these chemoattractants.^16^ Indeed, previous reports have shown that IL-17A-driven neutrophil transmigration (through TNF-α-stimulated murine ECs or resting human lung microvascular ECs in vitro) was completely abolished when chemokine receptor (CXCR)2^-/-^ neutrophils were perfused over the cultures^23^ or when cultures were treated with neutralising antibodies against CXCL8/IL-8.^27^ We further corroborated this idea through proteome analysis of cyto-chemokines released locally in response to either IL-17A or nIL-17 (figure 2C–F). Importantly, nIL-17 augments the amount of several pro-inflammatory mediators, such as IL-8/KC, MCP-1/JE, soluble intercellular adhesion molecule 1 (sICAM1),^28^ when compared with native IL-17A (figure 2F). Others have reported similar IL-17-induced increases in inflammatory mediators (KC, JE, interferon gamma (IFN-γ), IL-1α, sICAM1, macrophage inflammatory proteins and IL-6) within a variety of tissues, including pouch cavities, brain, blood and aorta.^16 29 30^ Taken together, these data reinforce our earlier observations that nIL-17 is a more potent pro-inflammatory stimulus than native full-length IL-17A protein and confirm that this peptide truly represents the most biological active sequence of this cytokine.

### nIL-17 amplifies EC activation to support leucocyte trafficking

IL-17 receptors are expressed by both haematopoietic cells of the immune system and by stromal cells, such as HDBEC.^27 31^ IL-17A synergistically amplifies HDBEC response to TNF-α, further increasing expression of the adhesion molecules ICAM-1 and vascular cell adhesion molecule-1 (VCAM-1).^23 27^ Given this, the effects we describe above for nIL-17 could be due to a direct effect on the leukocytes or the HDBEC alone or on both cell types. To address this, we analysed the adhesion (phase bright) and transmigration (phase dark) of peripheral blood mononuclear cells (PBMC) across inflamed ECs following the addition of IL-17A or nIL-17 (figure 2G). As we have previously published,^32^ TNF-α stimulation enhanced PBMC adhesion, but this was not further amplified in the presence of either IL-17A protein or nIL-17 peptide (figure 2H). In agreement with our findings, IL-17 did not enhance absolute numbers of adherent leucocytes (neutrophils) to TNF-α stimulated endothelium in vitro or in vivo, but rather altered cellular behaviour, increasing the number undergoing transmigration.^23^ Like earlier findings, nIL-17 exacerbated PBMC migration through inflamed endothelium to the same extent as seen with full-length IL-17A (figure 2I). PBMC capture and firm adhesion are mediated through E-selectin and VCAM-1 expressed by the inflamed endothelium,^32^ with VCAM-1 levels remaining unaffected by the addition of either IL-17A protein or peptide in combination with TNF-α when compared with TNF-α alone (figure 2J) in agreement with previous publications.^23^ Of note, neither IL-17A protein nor peptide induced VCAM-1 expression in the absence of TNF-α. By contrast, PBMC transmigration is dependent on β_2_-integrins binding endothelial ICAM-1,^33^ with the protein for the latter being synergistically elevated by both IL-17A or nIL-17 (figure 2K). No differences were observed in IL-17RA or RC expression on ECs under any conditions used (online supplemental figure S8A,B). These findings demonstrate that nIL-17 enhances endothelium activation in response to inflammation, to further amplify leucocyte migration.

### nIL-17 specific antibody (Ab-IPL-IL-17) displays potent neutralising activity

**Figure 3:**
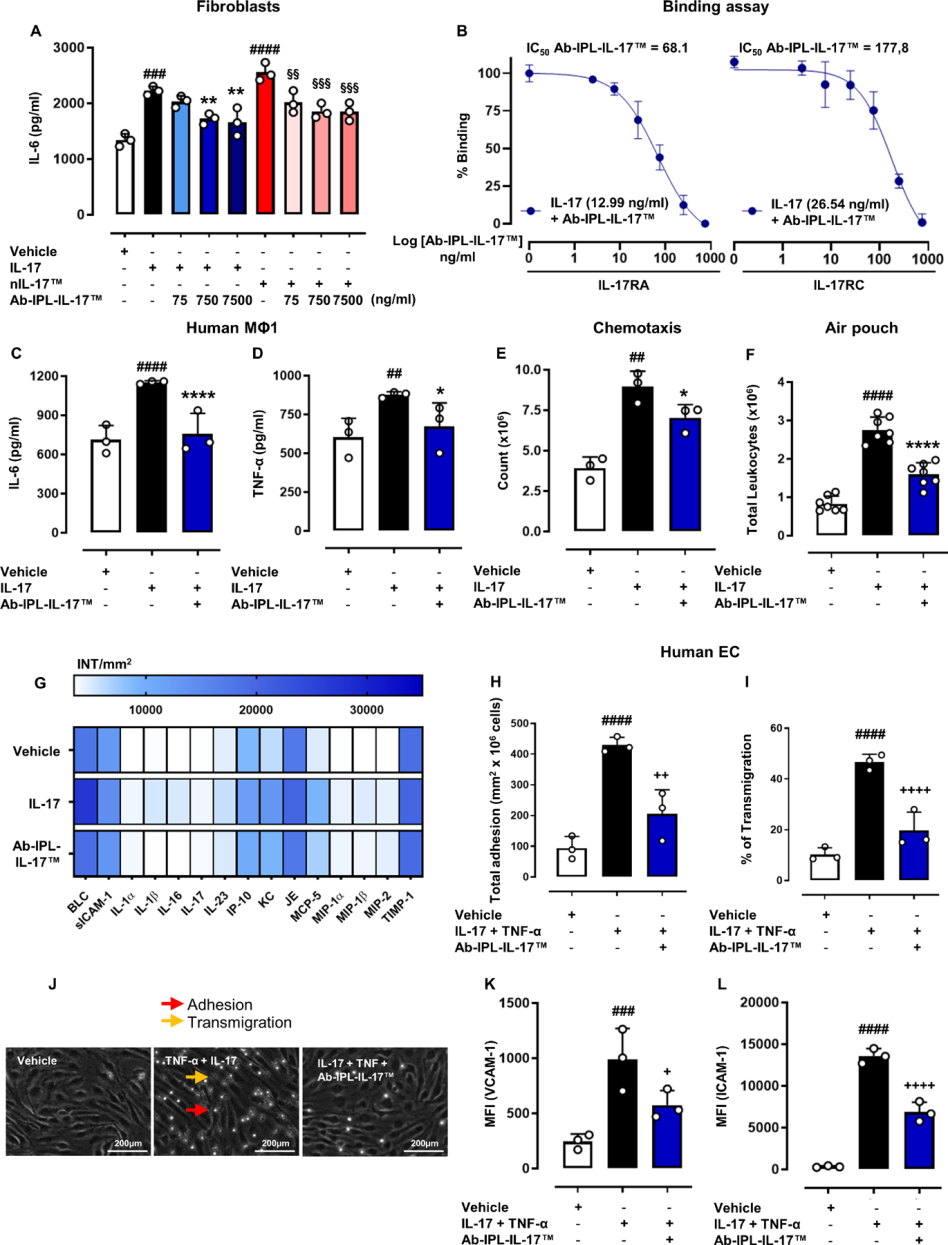
Biological characterisation of a novel IL-17 neutralising antibody (Ab-IPL-IL-17). (A) To assess the biological activity of Ab-IPL-IL-17, IL-6 production was evaluated in NIH-3T3 cell supernatants following 24 hours treatment with IL-17 (50 ng/mL) or nIL-17 (50 ng/mL) alone or in combination with Ab-IPL-IL-17 (75–750 ng/mL). (B) To analyse the neutralisation effect of Ab-IPL-IL-17 on IL-17/IL-17Rs interactions, biotinylated IL-17 (EC_50_ concentrations) and Ab-IPL-IL-17 (0–750 ng/mL) complex was co-incubated for 30 min with IL-17RA-Fc or IL-17RC-Fc prior to fluorescence being measured. (A)–(B) Data are presented as mean±SD of n=3 independent experiments. (C)–(D) Macrophages, derived from primary human CD14^+^ monocytes, were stimulated with LPS and IFN-γ (M1-stimuli) over 16 hours. Following differentiation, cells were treated with IL-17 vehicle, IL-17 (100 ng/mL) alone or in combination with Ab-IPL-IL-17 (10 µg/mL) for 24 hours. Supernatants from all experimental conditions were assayed by ELISA for (C) IL-6 and (D) TNF-α. (E) For the transwell chemotaxis assay, neutrophils were added to the top chamber, which had a confluent stimulated (TNF-α and IFN-γ) HDBEC monolayer. (E) Chemotactic migration to IL-17 (500 ng/mL) alone or in combination with Ab-IPL-IL-17 (10 µg/mL) was quantified using flow cytometry. (C)–(E) Data are presented as means±SD of n=3 independent healthy donors. (F) For in vivo experiment, mice were treated with IL-17 vehicle (0.5% CMC), IL-17 (1 µg/pouch) alone or in co-administration with Ab-IPL-IL-17 (10 µg/mL), and thereafter total CD45^+^ leucocyte numbers were quantified by flow cytometry. (F) Data are presented as means±SD of n=7 mice per group. (G) Inflammatory supernatants obtained from the pouch cavities were assayed using a Proteome Profiler Cytokine Array. Densitometric analyses are presented as a heat map indicating the most significant modulated cyto-chemokines mediators. (G) Data are presented as means±SD of positive spots of three separate independent experiments run each with n=7 mice per group pooled. (H)–(J) HDBECs were treated with IL-17 vehicle (HCl 4 mM PBS), IL-17 (100 ng/mL) plus TNF-α (100 U/mL) alone or in combination with Ab-IPL-IL-17 (10 µg/mL) for 24 hours. Phase bright PBMCs were considered (H) adherent (red arrow), whereas phase-dark were quantified as (I) transmigrated (% of adherent cells) (orange arrow). (J) Representative images of the static adhesion assay are shown (200 µm magnification). (K)–(L) VCAM-1 and ICAM-1 expression on HDBECs was quantified by flow cytometry. (H)–(L) Data are presented as means±SD of n=3 independent healthy donors. Statistical analysis was conducted by one or two-way analysis of variance test followed by Bonferroni’s for multiple comparisons. ^##^p≤0.01, ^###^p≤0.001, ^####^p≤0.0001 vs vehicle group; *p≤0.05, **p≤0.01, ****p≤0.0001 vs IL-17 group; ^§§^p≤0.01, ^§§§^p≤0.001, ^§§§§^p≤0.0001 vs nIL-17 group; ^+^p≤0.05, ^++^p≤0.01, ^++++^p≤0.0001 vs IL-17+TNF-α group. CMC, carboxymethyl cellulose; HDBECs, human dermal blood endothelial cells; ICAM-1, intercellular adhesion molecule-1; IFN-γ, interferon gamma; IL-17, interleukin-17; LPS, lipopolysaccharide; PBMCs, peripheral blood mononuclear cells; PBS, phosphate-buffered saline; TNF-α, tumour necrosis factor α; VCAM-1, vascular cell adhesion molecule-1.

Given that nIL-17 clearly demonstrates a more prominent inflammatory activity than full-length IL-17A/F, we embarked on generating a novel IL-17 neutralising antibody: Ab-IPL-IL-17 targeting the nIL-17 sequence (IT patent no.102022000016722). Ab-IPL-IL-17 significantly decreased IL-6 production from (commercially available) IL-17A homodimer and nIL-17-stimulated NIH-3T3 cells in a concentration-dependent manner (figure 3A) without any cytotoxic effect for all tested concentrations on murine embryonic fibroblast cell lines (online supplemental figure S9); reduced binding affinity of IL-17A to IL-17RA or RC (figure 3B); blocked the production of IL-6 and TNF-α from IL-17A-treated M1 macrophages (figure 3C,D, respectively) and decreased IL-17A-induced neutrophil migration (figure 3E). In vivo, Ab-IPL-IL-17 simultaneously administered with IL-17A reduced influx of total CD45^+^ leucocytes into the inflamed air pouch (figure 3F), with a corresponding reduction observed in several cyto-chemokines (figure 3G). Similar observations were made in vitro, where pretreatment with Ab-IPL-IL-17 significantly reduced both PBMC and peripheral blood lymphocyte adhesion to and transmigration through IL-17 + TNF-α treated endothelium (figure 3H–J and online supplemental figure S10, respectively), which was mirrored by decreased HDBEC expression of VCAM-1 and ICAM-1 (figure 3K,L).

### Ab-IPL-IL-17 maintains activity in the absence of off-target immunogenic effects as seen with secukinumab

**Figure 4:**
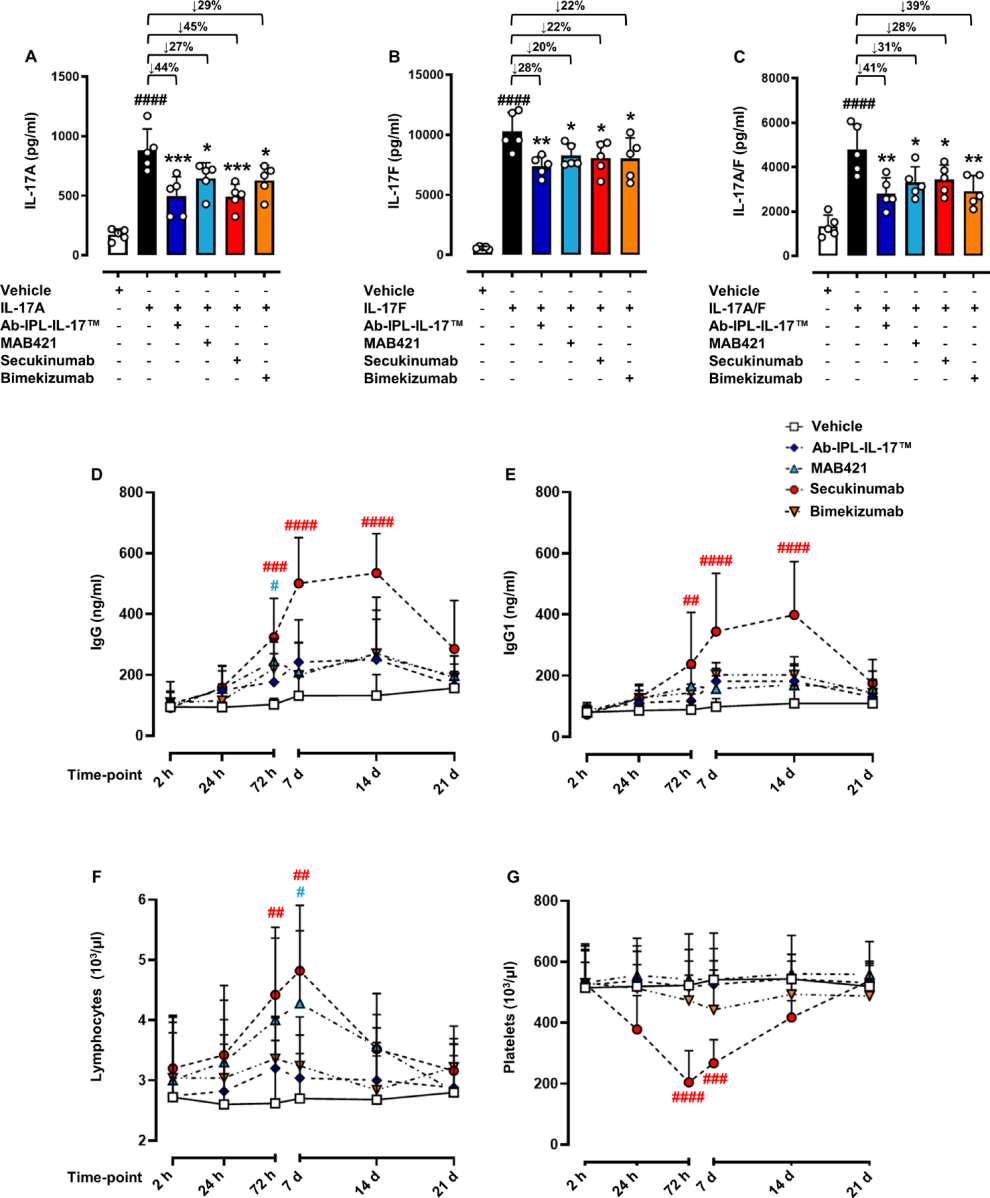
Ab-IPL-IL-17 displays a protective profile in murine preclinical models of immune-mediated inflammatory diseases. To assess the neutralising activity of Ab-IPL-IL-17, CD-1 mice were injected i.p. with 100 µg/mouse of Ab-IPL-IL-17, MAB421, secukinumab or bimekizumab as positive controls. After 30 min, an i.p. injection of 10 µg/mouse of IL-17A, IL-17F or IL-17A/F heterodimer was administered. After 2hours, blood was collected by intracardiac puncture and serum levels of (A) IL-17A, (B) IL-17F or (C) IL-17A/F were quantified by ELISA. (D)–(G) For the evaluation of immunogenic effects, CD-1 mice were injected i.p. with 100 µg of IgG1 isotype antibody (vehicle) or IL-17 neutralising antibodies (secukinumab, bimekizumab, MAB421 or Ab-IPL-IL-17). In the selected time-point (2 hours, 24 hours, 72 hours, 7 days, 14 days and 21 days), (D) total IgG, (E) IgG1, (F) lymphocytes and (G) platelets levels were determined by ELISA and haematological blood count test, respectively. Data are presented as mean±SD for n=5 mice per group. Statistical analysis was conducted by one or two-way analysis of variance test followed by Bonferroni’s for multiple comparisons. *p≤0.05, **p≤0.01, ***p≤0.001 vs IL-17 group; ^#^p≤0.05, ^##^p≤0.01, ^###^p≤0.001, ^####^p≤0.0001 vs vehicle group (in red refers to secukinumab and in light blue refers to MAB421, respectively). IgG, immunoglobuin G; IL-17, interleukin 17; i.p., intraperitoneal.

Current anti-IL-17A therapies (secukinumab and ixekizumab) are associated with unwanted off-target immunogenic effects, lymphocytosis and thrombocytopenia.^34^ As such, numerous clinical trials are currently investigating new biologic therapies targeting IL-17 biology/function to improve clinical outcomes in patients with IMIDs.^35^ Here, we evaluated in vivo the neutralising potential of Ab-IPL-IL-17 on IL-17A, IL-17F and IL-17A/F heterodimer production. Ab-IPL-IL-17 was able to significantly reduce the plasma concentration of IL-17A and IL-17F to similar levels as seen with secukinumab (figure 4A,B). Notably, Ab-IPL-IL-17 significantly reduced the plasma concentration of IL-17A/F heterodimer to a similar extent as the reference anti-IL-17A/F antibody bimekizumab (figure 4C). To assess immunogenicity under homeostatic conditions, we administered a single dose of Ab-IPL-IL-17 and measured total immunoglobulin G (IgG) and IgG1 levels over 21 days, comparing levels with the reference anti-IL-17 antibody (MAB421) and the current gold standard clinical therapies secukinumab and bimekizumab (figure 4D,E). A significant increase in total IgG and IgG1 was observed at 72 hours and remained elevated until day 14 with secukinumab, but no increase was observed in mice injected with Ab-IPL-IL-17 (figure 4D,E) or bimekizumab treatment. Whole blood analysis revealed that secukinumab and MAB421 increased total circulating lymphocyte numbers 72 hours post-injection/administration, which remained significantly elevated up to 7 days when compared with vehicle control (figure 4F). Furthermore, secukinumab induced thrombocytopenia as early as 24 hours and platelet numbers remained significantly reduced at 72 hours and 7 days post administration (figure 4G). Strikingly, Ab-IPL-IL-17 (and the selective IL-17A/F neutralising antibody bimekizumab) had no effect on total lymphocyte or platelet numbers at any time point assessed (figure 4F,G). No changes were observed for other haematological parameters (table 1). Collectively, these data show that Ab-IPL-IL-17 retains strong neutralising activity without triggering unwanted immunogenic response, making it an attractive clinical therapy.

### Ab-IPL-IL-17 reduces pathological symptoms of arthritis and IBD

**Figure 5:**
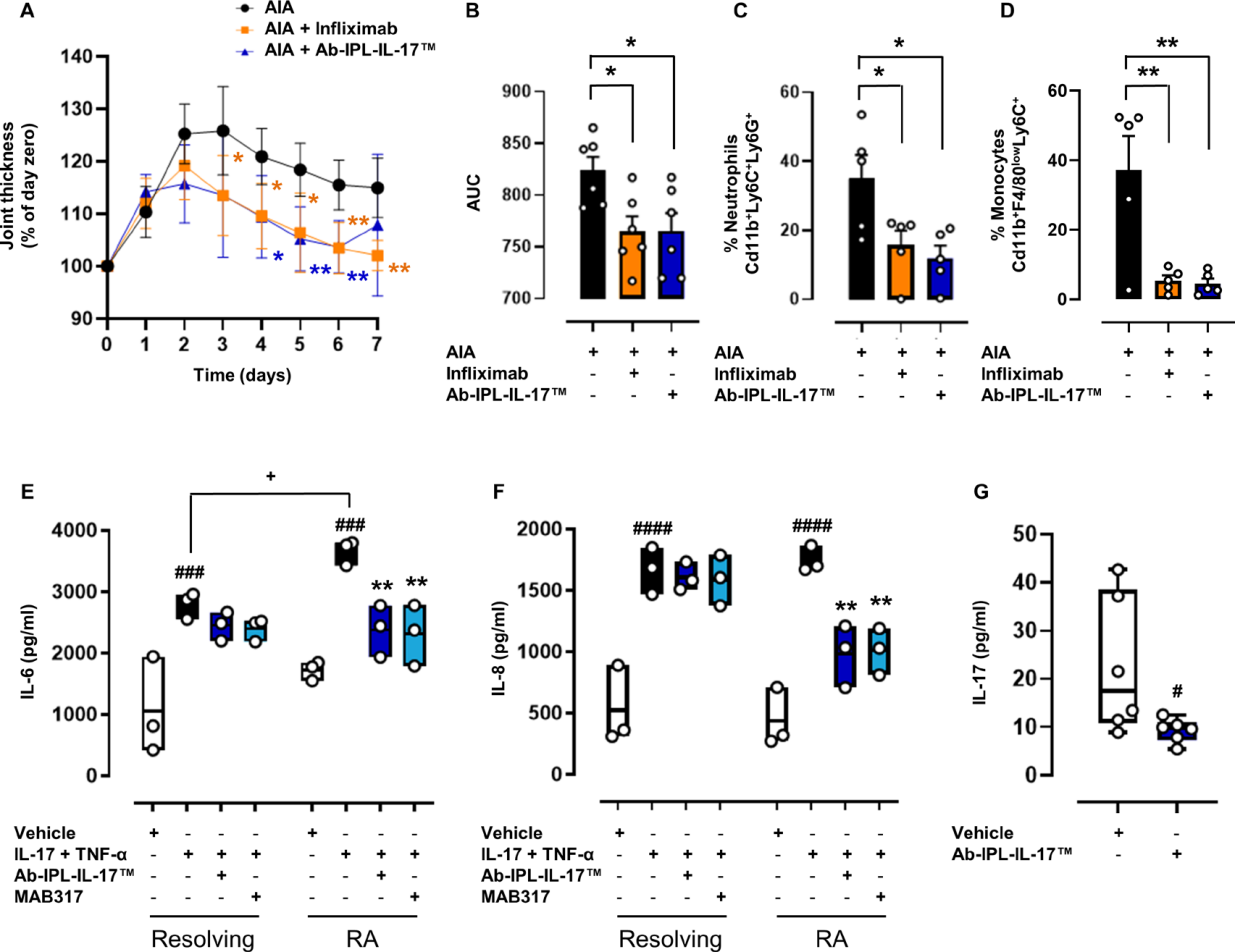
Ab-IPL-IL-17 displays a protective profile in human preclinical models of IMIDs. Monoarthritic mice (AIA group) were therapeutically administered Ab-IPL-IL-17 or infliximab (anti-TNF-α) on day 1 and day 3. Joint inflammation was scored daily and expressed as (A) percentage of baseline joint thickness or (B) AUC. Flow cytometry analysis was employed to determine in situ neutrophil and monocyte levels. At 7-day time-point, ankle joints were digested, and total cells were gated, followed by single cells, before the identifications of CD45. CD45^+^ cells were plotted to identify the % of CD11b^+^/LY6C^+^/LY6G^+^ as neutrophils (C) and CD11b^+^/F4/80^low^/LY6C^+^ as monocytes (D). Data are presented as mean±SD for n=5–6 mice per group. Statistical analysis was conducted by one or two-way analysis of variance test followed by Dunnett post-test. *p≤0.05, **p≤0.01 vs AIA group. (E)–(F) Fibroblasts from patients with resolving arthritis or RA were treated with IL-17 (10 ng/mL) and TNF-α (100 U/mL) alone or in combination with MAB317 or Ab-IPL-IL-17 (10 µg/mL). Secretion of (E) IL-6 or (F) IL-8 were measured by ELISA. Data are median±IQRs (min. 25%, max. 75%) for n=3 independent donors. (E)–(F) Statistical analysis was conducted by one-way ANOVA followed by Bonferroni’s for multiple comparisons. ^###^p≤0.001, ^####^p≤0.0001 vs own vehicle group; **p≤0.01 vs own IL-17+TNF-α; ^+^p≤ 0.05 vs resolving IL-17+TNF-α. (G) Human whole blood from patients with IBD was treated with or without Ab-IPL-IL-17 (10 µg/mL) for 4 hours, after which serum IL-17 levels were assessed by ELISA assay. Data are median±IQRs (min. 25%, max. 75%) for n=6 independent donors. Statistical analysis was conducted by one-way analysis of variance test followed by Bonferroni’s for multiple comparisons. ^#^p≤0.05 vs vehicle group. AIA, antigen-induced arthritis; AUC, area under the curve; IBD, inflammatory bowel disease; IL-17, interleukin 17; IMIDs, immune-mediated inflammatory diseases; RA, rheumatoid arthritis; TNF-α, tumour necrosis factor α.

Secukinumab and ixekizumab are current therapies for PsA and AS; therefore, we investigated the clinical efficacy of Ab-IPL-IL-17 in preclinical murine models of arthritis and ex vivo analysis from tissues of patients with RA or IBD. Excitingly, we found that therapeutic administration of Ab-IPL-IL-17 significantly reduced joint swelling in the murine antigen-induced arthritis (AIA) model (figure 5A,B). Indeed, treating AIA with commercially available neutralising antibodies to IL-17 has been previously reported to reduce clinical symptoms of arthritis^36^ and neutrophil accumulation within the joint.^37^ It is important to note that Ab-IPL-IL-17 was as effective at halting disease progression and triggering resolution as the gold-standard current treatment for RA, infliximab (figure 5A,B) with a significant reduction in infiltrating neutrophils (figure 5C). and monocytes (figure 5D) observed.

As a proof-of-concept of our investigation, we next analysed the potential clinical benefit of Ab-IPL-IL-17 in the treatment of patients with RA and IBD using ex vivo analysis of patient materials ensuring an equal proportion of male and female donors. First, we tested the actions of Ab-IPL-IL-17 on fibroblasts obtained from treatment naïve patients with acutely resolving arthritis or persistent RA. These isolated cells constitutively release IL-6 and IL-8 when in culture, and this was significantly increased when the fibroblasts were stimulated with recombinant IL-17 and TNF-α (figure 5E,F). Others have shown IL-17-induced increases in IL-6 and IL-8 gene expression and secretion from fibroblasts isolated from patients treated with RA undergoing joint replacement surgery.^38^ In agreement with our data, a combination treatment of IL-17 with TNF-α amplified IL-8 gene expression, but in contrast with our findings no changes in IL-6 were observed. Ab-IPL-IL-17 and the reference function blocking anti-IL-17 antibody (MAB317) were able to reverse this effect in inflamed RA fibroblasts to a similar degree, such that significantly lower concentrations of IL-6 and IL-8 were released (figure 5E,F). By contrast, neither MAB317 nor Ab-IPL-IL-17 altered the IL-6 or IL-8 secretion from IL-17 + TNF-α activated resolving fibroblasts (figure 5E,F). These data strongly indicate the Ab-IPL-IL-17 therapy specifically inhibits the pro-inflammatory actions of chronically inflamed fibroblasts within the rheumatoid joint but does not adversely alter the protective response elicited during acutely resolving joint inflammation. In the context of IBD, treatment with several IL-17 neutralising antibodies has been shown to have limited efficacy with some patients completely refractory to treatment.^39^ In a proof-of-concept experiment, Ab-IPL-IL-17 was able to deplete/sequester plasma IL-17A within samples obtained from treatment naïve patients with IBD attending an inception clinic (figure 5G) but had no effect on plasma IL-6 or TNF-α concentrations (online supplemental figure S11). These data indicate that Ab-IPL-IL-17 has the potential to effectively alleviate pathological pro-inflammatory (IL-17s-mediated) responses in patients with IMIDs.

## Discussion

While current biologics targeting IL-17A/F exist for the treatment of various IMIDs, issues with immunogenicity, partial/incomplete patient responses and adverse side effects are driving the field to design and develop more effective biologics.^5^ Here, we initially identified a bioactive 20-mer IL-17A/F-derived peptide (nIL-17) that mimics the pro-inflammatory actions of the full-length proteins. Subsequently, we generated a novel anti-IL-17 neutralising monoclonal antibody (Ab-IPL-IL-17) capable of effectively reversing the pro-inflammatory, pro-migratory actions of nIL-17. Importantly, we demonstrated that in mice Ab-IPL-IL-17 has less haematological off-target effects than the current gold-standard biologics. Finally, we found that Ab-IPL-IL-17 effectively reduced clinical signs of experimental arthritis, decreased in vitro pro-inflammatory cytokine production by synovial fibroblast cells and neutralised elevated IL-17 levels in IBD patient serum following ex-vivo stimulation. Collectively, our preclinical and in vitro clinical evidence indicates high efficacy and therapeutic potency of Ab-IPL-IL-17, supporting the rationale for large-scale clinical evaluation of Ab-IPL-IL-17 in patients with IMIDs.

Currently, numerous therapeutics targeting IL-17/IL-17R pathway for the treatment of IMIDs are available, including several approved monoclonal antibodies (secukinumab and ixekizumab) and several newer biologics under clinical trials, such as bimekizumab.^8 13 40^ However, some of these have been discontinued in certain patient groups due to severe/damaging side effects. Increased intestinal inflammation has been reported in patients with IBD treated with secukinumab or brodalumab, thus increasing disease severity.^41^ Similarly, various IL-17 biologics have been associated with increased numbers of *Candida* or upper respiratory tract infections in numerous different patient groups, further increasing patient morbidity.^40 42^ Psoriasis patients treated with brodalumab have experienced depression/anxiety linked with suicidal thoughts.^43^ It is well accepted within the field that stringent pharmacovigilance measures are required to ascertain drug safety and adverse risk events.

The short antigen recognition sequence (approximately six times shorter) of Ab-IPL-IL-17 offers a significant clinical advantage over other known anti-IL-17 monoclonal antibodies, by reducing the incidence of non-specific binding that can result from longer amino acid sequences.^44^ Moreover, short peptide sequences also offer lower production costs and manufacturing advantages when compared with large protein targets. Of potential clinical importance, Ab-IPL-IL-17 displays, in mice, equivalent efficacy as reference and gold-standard current treatment and commercially available anti-IL-17 neutralising antibodies but crucially displays reduced immunogenicity and haematological side effects, that are major issues currently faced by patients.^8 13^

In conclusion, we have identified the bioactive sequence of IL-17A/F that is responsible for driving inflammation, which has conserved sequence homology in mice and humans. Using this unique sequence, we have generated a specific cross-species neutralising antibody allowing discovery in vitro and pre-clinical assessment, as well as having the potential to be translated directly into clinic as a new therapy. Crucially, Ab-IPL-IL-17 has no immunogenicity, lymphocytosis or thrombocytopenia properties, highlighting its clinical superiority over current therapies, including secukinumab. Future studies and clinical trials will need to address the varying requirements of Ab-IPL-IL-17 as an alternative biological therapy for treating patients with IMIDs.

## Materials and methods

### Animals

Experiments were carried out in 8–12-week-old male CD-1 mice according to the guidelines for the safe use and care of experimental animals in accordance with the Italian D.L.no.116 of 27 January 1992 (500/2020-PR and 507/2022-PR) and associated guidelines in the European Communities Council (86/609/ECC and 2010/63/UE), including the 3Rs concept.^45^ Animals were housed with ad libitum access to food and water and maintained on a 12-hour light/dark cycle. Experimental study groups were randomised and blinded. All procedures were carried out to minimise the number of animals used (n=5–7 per group) and their suffering.

### Air pouch

Dorsal air pouches were prepared by injection of 2.5 mL of air on day 0 and day 3 in CD-1 mice, as previously described.^25^ On day 6, mice received 0.25 mL of one of the following treatments diluted in 0.5% carboxymethyl cellulose (CMC, Sigma-Aldrich): (1) vehicle, CMC alone; (2) IL-17 (1 µg); (3) nIL-17 (1 µg); (4) IL-17 (1 µg) plus MAB421 or Ab-IPL-IL-17 (10 µg); (5) IL-17 (1 µg) plus anti-JE (10 µg, MAB479, R&D System) and (6) IL-17 (1 µg) plus anti-KC (10 µg, MAB453, R&D System). Mice were sacrificed after 24 hours, and lavage fluids were recovered, and centrifuged at 220 g for 10 min at 4°C. Cell pellets and inflammatory exudates were banked for subsequent analysis of inflammatory cyto-chemokines. The route, timing and frequency of administration as well as the selected dosages of tested compounds were selected according to updated literature.^12 16^ Cell number was determined by TC20 automated cell counter (Bio-Rad) using Bio-Rad’s TC20 automated cell counter uses disposable slides, TC20 trypan blue dye (0.4% trypan blue dye w/v in 0.81% sodium chloride and 0.06% potassium phosphate dibasic solution, Sigma-Aldrich) and a CCD camera to count cells based on the analyses of capture images.^25^

### AIA model

Animal studies were regulated by the Animals (Scientific Procedures) Act 1986 of the UK and performed under appropriate Personal Project License. Approval was granted by the University of Birmingham’s Animal Welfare and Ethical Review Body and all ethical guidelines were adhered to while carrying out this study. Eight-week-old male, C57Bl/6J wild-type mice were purchased from Charles River and were maintained in a specific pathogen free facility, with free access to food and water. Environmental conditions were: 21±2 °C, 55%±10% relative humidity and a 12-hour light–dark cycle. Mice were immunised with methylated bovine serum albumin (mBSA, 10 µg subcutaneous, Sigma-Aldrich) in complete Freund’s adjuvant (Thermofisher scientific, Milan, Italy).^46^ On day 21, monoarthritis was induced by intra-articular injection of mBSA (100 µg) into the knee. Mice were treated therapeutically at 24 hours or 72 hours post disease onset by intraperitoneal injection with 50 µg of either infliximab (anti-TNF-α) or a neutralising antibody to IL-17 (Ab-IPL-IL-17). Joint thickness (mm) was measured by callipers daily for up to 7 days. Data are expressed as a percentage change from baseline measurement taken on day 21 or area under the curve.

### Human blood samples

Whole blood was collected in EDTA-coated vacutainers from healthy donors and patients with IBD^47–49^ with written informed consent. An equal proportion of male and female donors were used with an age range between 22 years and 70 years. (Tables 2 and table 3) (please also refer to online supplemental materials and methods).

**Table 2:** Demographic, clinical and laboratory characteristics of patients in each outcome group

	Resolving (n=3)	RA (n=3)
Age (years)*	41 (27–87)	56 (49–62)
Female; n (%)	2 (67)	3 (100)
Symptom duration (weeks)*	4 (4–7)	260 (104–1052)
DAS28 ESR at baseline†	4.1±2.5	5.7±0.5
ESR (mm/hour)*	37 (5–60)	22 (13–63)
CRP (mg/L)*	28 (9–52)	62 (3–67)
RF positive (%)	0 (0)	3 (100)
ACPA positive (%)	0 (0)	2 (100)
SJC28*	2 (2–11)	9 (4–11)
TJC28*	3 (0–16)	9 (4–11)
VAS*	10 (3–50)	40 (32–73)
US GS*	2 (1–2)	–
US PD*	0 (0–2)	–
NSAIDs (%)	3 (100)	2 (67)

*Median (IQR).

†Mean±SD.

-, data not obtained from patients at time of presentation; ACPA, anti citrullinated protein antibody; CRP, C reactive protein; ESR, erythrocyte sedimentation rate; NSAIDs, non-steroidal anti-inflammatory drugs; RA, rheumatoid arthritis; RF, rheumatoid factor; SJC28, 28 swollen joint count; TJC28, 28 tender joint count; US GS, ultrasound greyscale grade at the biopsied joint; US PD, ultrasound power Doppler grade at the biopsied joint; VAS, Visual Analogue Scale.

**Table 3:** Demographic, clinical and laboratory characteristics of patients with IBD

	UC (n=2)	CD (n=2)	IBS (n=2)	Metastatic colorectal cancer (n=1)
Age (years)*	31 (26–36)	36.50 (33–40)	22 (19–25)	44
Female: number (%)	0 (0)	1 (50)	1 (50)	1 (100)
Ethnicity Asian: n (%)	1 (50)	2 (100)	0 (0)	1 (100)
Symptom duration (months)*	2 (1–3)	5 (3–7)	24 (12–36)	2
Weight (kg)*	92.70 (76.70–108.70)	85.75 (66.50–105)	67.05 (59.80–74.30)	95.70
Height (m)*	1.77 (1.75–1.80)	1.68 (1.58–1.79)	1.73 (1.67–1.80)	1.65
BMI*	29.30 (25–33.60)	29.70 (26.60–32.80)	22.15 (21.40–22.90)	35.20
Smoker: n (%)	0 (0)	0 (0)	1 (50)	1 (100)
FCAL at baseline (μg/g)†	2302±0	750±963.1	44.50±34.65	583±0
CRP (mg/L)*	13.5 (3–24)	2.50 (1–4)	2 (1–3)	7
HBI*	na	10 (9–11)	na	na
Partial Mayo*	5 (4–6)	na	na	na
Endoscopic Mayo*	2 (2–2)	na	na	na
SES-CD*	Na	3 (3–3)	na	na
Montreal classification	A2E3S2	A2L1B1	na	na
Treatment (initiated after sampling)	Mesalazine Mesalazine Budesonide Azathioprine	Budesonide	–	–

Categorising race and ethnicity=white and Asian.

*Median (IQR).

†Mean±SD.

-, data not obtained from patients at time of presentation; BMI, body mass index; CD, Crohn’s disease; CRP, C reactive protein; FCAL, faecal calprotectin; HBI, Harvey-Bradshaw Index; IBS, irritable bowel syndrome; na, not applicable; SES-CD, Simple Endoscopic Score for Crohn’s disease; UC, ulcerative colitis.

### Statistical analysis

Statistical analysis complies with the international recommendations on experimental design, analysis and data sharing and presentation in preclinical pharmacology.^30 50^ Data are presented as mean±SD or median±IQR. Normality was tested prior to analysis with one or two-way ANOVA followed by Bonferroni’s or Dunnett’s for multiple comparisons, where p ≤0.05 was deemed significant. Animal weight was used for randomisation and group allocation to reduce unwanted sources of variations by data normalisation. No animals and related ex vivo samples were excluded from the analysis. In vivo study was carried out to generate groups of equal size (n=5–7 of independent values), using randomisation and blinded analysis.

### Experimental procedures and materials

Murine and human cell lines and culture, ex vivo whole blood assay, ELISA-based binding assay, transwell migration assay, ex vivo analysis, haematological investigations, western blot analysis, flow cytometry, synthesis of a novel IL-17 neutralising antibody, immunisation and fusion protocols and computational studies are described in online supplemental materials and methods.

### Data, materials and software availability

All data associated with this study are present in the paper or the online supplemental materials. Requests for reagents (antibodies and other proteins) should be directed to the corresponding authors and will be made available after completion of a material transfer agreement with ImmunoPharmaLab, Department of Pharmacy, University of Naples Federico II.

## Data Availability

Data are available upon reasonable request. No data are available. All data associated with this study are present in the paper or the online supplemental materials. Requests for reagents (antibodies and other proteins) should be directed to the corresponding authors and will be made available after completion of a material transfer agreement with ImmunoPharmaLab, Department of Pharmacy, University of Naples. Federico II.
