# Supplementary material for: New biologic (Ab-IPL-IL-17) for IL-17-mediated diseases: identification of the bioactive sequence (nIL-17) for IL-17A/F function
Source: Ann Rheum Dis. 2023 Aug 14;82(11):1415–28. doi: 10.1136/ard-2023-224479 (PMC10579190; doi:10.1136/ard-2023-224479)
Supplement: Supplementary data [file ard-2023-224479supp001.pdf]

Supplementary Information for

New biologic (Ab-IPL-IL17™) for IL-17-mediated diseases: Identification of the bioactive sequence (nIL-17™) for IL-17A/F function.

Anella Saviano[1,†], Adel Abo Manosour[2†], Federica Raucci[1,†], Francesco Merlino[3], Noemi Marigliano[1], Anna Schettino[1], Mussarat Wahid[4], Jenefa Begum[5], Andrew Filer[4], Julia E Manning[4], Gian Marco Casillo[3], Marialuisa Piccolo[6], Maria Grazia Ferraro[6], Simona Marzano[3], Pasquale Russomanno[3], Rosa Bellavita[3], Carlo Irace[6], Jussara Amato[3], Mohammed Alfaifi[2], Peter Rimmer[5,7], Tariq H Iqbal[7,8], Stefano Pieretti[9], Valentina Vellecco[3], Francesco Caso[10], Luisa Costa[10], Roberto Giacomelli[11], Raffaele Scarpa[10], Giuseppe Cirino[3], Mariarosaria Bucci[3], Helen M McGettrick[4,‡], Paolo Grieco[3,‡], Asif Jilani Iqbal[5,1,‡,*] and Francesco Maione[1,‡,*].

[1]*ImmunoPharmaLab*, Department of Pharmacy, School of Medicine and Surgery, University of Naples Federico II, Via Domenico Montesano 49, 80131, Naples, Italy.

[2]Department of Clinical Laboratory Sciences, College of Applied Medical Sciences, King Khalid University, Abha, Saudi Arabia.

[3]Department of Pharmacy, School of Medicine and Surgery, University of Naples Federico II, Via Domenico Montesano 49, 80131, Naples, Italy.

[4]Institute of Inflammation and Ageing (IIA), College of Medical and Dental Sciences, University of Birmingham, Birmingham, B15 2WB, UK.

[5]Institute of Cardiovascular Sciences (ICVS), College of Medical and Dental Sciences, University of Birmingham, Birmingham, B15 2TT, UK.

[6]*BioChemLab*, Department of Pharmacy, School of Medicine and Surgery, University of Naples Federico II, Via Domenico Montesano 49, 80131, Naples, Italy.

[7]Department of Gastroenterology, Queen Elizabeth Hospital Birmingham NHS Foundation Trust, Birmingham, UK.

[8]Institute of Microbiology and Infection (IMI), College of Medical and Dental Sciences, University of Birmingham, Birmingham, B15 2TT, UK.

[9]National Center for Drug Research and Evaluation, Istituto Superiore di Sanità, Viale Regina Elena 299, 00161, Rome, Italy.

[10]Rheumatology Research Unit, Department of Clinical Medicine and Surgery, University of Naples Federico II, via S. Pansini 5, 80131 Naples, Italy.

[11]Clinical Unit of Rheumatology and Clinical Immunology, School of Medicine, University of Rome "Campus Biomedico", 00128, Rome, Italy.

[†]These authors share first authorship.

[‡]These authors share senior authorship.

Saviano A, *et al. Ann Rheum Dis* 2023; 82:1415–1428. doi: 10.1136/ard-2023-224479

***Author for correspondence: Francesco Maione**, Head of *ImmunoPharmaLab*, Department of Pharmacy, School of Medicine, University of Naples Federico II, Via Domenico Montesano 49, 80131, Naples, Italy. E-mail: francesco.maione@unina.it. **Asif Jilani Iqbal**, Institute of Cardiovascular Sciences (ICVS), College of Medical and Dental Sciences, University of Birmingham, Birmingham, B15 2TT, UK. E-mail: a.j.iqbal@bham.ac.uk

Supplementary Materials and Methods

Cell culture

Mouse embryonic fibroblast cell line

Mouse embryonic fibroblast cell line (NIH-3T3) was cultured in 100 × 20 mm dishes (5×10^5 cells/dish) in DMEM (Sigma-Aldrich, Milan, Italy) supplemented with 10% fetal bovine serum (FBS; Sigma-Aldrich), 2 mM L-glutamine, 100 U/ml penicillin, 100 µg/ml streptomycin, 25 mM HEPES and 120 µg/ml Na pyruvate in a humidified 5% CO_2 at 37 °C. Cell viability was examined using a colorimetric assay based on the MTT labelling reagent. NIH-3T3 cells were seeded in 96-well plates and, after overnight incubation, were treated with nIL-17™ (7.5-750 ng/ml) or Ab-IPL-IL-17™ (7.5-7500 ng/ml). After 24 h, 10 µl of MTT solution (5 mg/ml in phosphate-buffered saline, PBS; pH 7.4) were added to each well and the plates were incubated for 3 h at 37 °C. Then, the medium was removed, and the obtained formazan crystals were dissolved in 150 µl of DMSO for 15 min. The spectrophotometric absorbance was measured using a microtiter enzyme-linked immunosorbent assay reader (Multiskan™ GO Microplate Spectrophotometer; Thermo Scientific™) at 540 nm. The percentage of cells viability was determined by the following formula: OD of treated cells/OD of vehicle × 100. For further *in vitro* experiments, following 70% confluency (1), NIH-3T3 were treated for 24 h with recombinant mouse (disulfide-linked homodimer) IL-17A (50 ng/ml, 421-ML, R&D System, Milan, Italy), (disulfide-linked homodimer) IL-17F (50 ng/ml, 2057-IL, R&D System) or heterodimer IL-17A/F (50 ng/ml, 5390-IL, R&D System) or nIL-17™ peptide (50 ng/ml) alone or in combination with IL-17 or nIL-17™ neutralising antibodies (MAB421 [R&D System] or Ab-IPL-IL-17™; 750 ng/ml). Supernatants were collected and stored at -80 °C for future use. Protein concentration was determined by the Bio-Rad protein assay (Bio-Rad, Milan, Italy) as previously described (2).

Human monocyte-derived macrophages (hMDMs) isolation and differentiation

PBMC were isolated from whole blood of healthy donors using histopaque (Sigma-Aldrich) density gradients as previously described (3). Mixed monocytes were isolated from the PBMC fraction, by positive selection using anti-CD14 microbeads (130-050-201, Miltenyi Biotec, Germany), in PBS without Ca^{2+} and Mg^{2+} supplemented with 0.5% BSA (Sigma-Aldrich) and 2 mM EDTA at 4 °C (Sigma-Aldrich), and MACS separation columns (Miltenyi Biotec). Purified monocytes (~95%) were cultured at 37 °C in 5% CO_2 in M199 media (Life Technologies, Paisley, U.K.) containing 10 ng/mL epidermal growth factor (EGF) (Sigma-Aldrich) and 10% autologous human serum (monocyte culture media) replaced every two days for six days (3). After six days, to induce “pro-inflammatory” (M1) or “pro-resolution” (M2) phenotypes, previously established protocols were followed (4). Briefly, M1 and M2 phenotypes were generated by treating human monocyte-derived macrophages (hMDMs) with lipopolysaccharide (LPS; 100 ng/mL; L4524-5MG, Sigma-Aldrich) and interferon gamma (IFN-γ, 20 ng/mL; 300-02, PeproTech, London, UK) or IL-4 (20 ng/mL; 200-04, PeproTech) respectively for 16 h. Then, cells, after polarisation, were stained for IL-17RA (clone 133617, R&D System) or IL-17RC (clone 309822, R&D System) or stimulated for 24 h with recombinant human IL-17 (317-ILB, R&D System), nIL-17™ (100 ng/ml) alone or in combination with neutralising antibodies MAB317 (R&D System), or Ab-IPL-IL-17™ at 10 µg/ml (5).

Human dermal blood endothelial cells (HDBEC)

HDBECs (PromoCell, Heidelberg, Germany) were cultured in 12-well plates in MV media (PromoCell) until confluent. HDBECs were stimulated for 24 h at 37°C with IL-17 or nIL-17™ (100 ng/ml) alone or in combination with TNF-α (100 U/ml, 210-TA, R&D System). In IL-17 neutralising antibodies experiments, cells were also pre-treated with neutralising antibodies MAB317 or Ab-IPL-IL-17™ (10 µg/ml) 30 min before stimulation with IL-17 or nIL-17™ (6, 7). Samples were analysed by flow cytometry or Elisa (see below).

Isolation of human peripheral blood lymphocytes (PBLs) and neutrophils

Venous blood from healthy volunteers was collected into 10 ml EDTA coated tubes. Peripheral blood mononuclear cells (PBMCs) were isolated by centrifugation of blood on histopaque 1077 (8), and PBL were prepared by panning human PBMC on culture plastic to remove monocytes (9, 10). Isolated cells were washed, counted, and adjusted to a final concentration of 1×10^6/ml in Medium 199, supplemented with 0.15% BSA (Sigma-Aldrich; M199BSA). On a few occasions, neutrophils were isolated using a two-step density gradient with histopaque 1077 and histopaque 1119 as previously described (11-13) and suspended at 1×10^6/ml in M199BSA, after hypotonic lysis of contaminating erythrocytes.

Isolation of human fibroblasts

Synovial tissue samples were obtained by ultrasound-guided biopsy (14) from treatment naïve patients with a new onset of clinically apparent arthritis and a symptom duration of ≤12 weeks who at follow-up had resolving arthritis (Res) (15). Patients were classified as having resolving arthritis if there was no clinical evidence of synovial swelling at any peripheral joint (out of a swollen joint count of 66 joints) on final examination at least one year after initial presentation, in

the absence of disease modifying anti-rheumatic drugs or glucocorticoid therapy for at least the previous 3 months (16). Alternatively, synovial tissue samples were collected from subjects with established, treated RA undergoing joint replacement surgery (RA). RA was classified according to 1987 American College of Rheumatology criteria (17) (**Table 2**). Fibroblasts, used between passages 4-6 (18), were isolated as previously described (19) and stimulated for 24 h at 37°C with IL-17 (10 ng/ml) in combination with TNF-α (100 U/ml). In IL-17 neutralising antibodies experiments, cells were also pre-treated with neutralising antibodies MAB317 or Ab-IPL-IL-17™ (10 µg/ml) 30 min before stimulation with IL-17 and TNF-α (20). Samples were analysed by Elisa (see below).

***Ex vivo* analysis**

***In vivo* neutralisation and immunogenicity assays**

For the neutralisation assay (21), CD-1 mice (n = 5 per group) were injected i.p. with 100 µg of IL-17 neutralising antibodies (secukinumab [Creative Biolabs, London, UK], bimekizumab [Creative Biolabs, UK] , MAB317 or Ab-IPL-IL-17™), 30 min prior to an i.p. injection of 10 µg IL-17A, IL-17F (2057-IL, R&D System) or heterodimer IL-17A/F (5390-IL, R&D System). 2 h after IL-17s administration, IL-17A, IL-17F and IL-17A/F levels were determined by Elisa. For the evaluation of immunogenic effects (22, 23) CD-1 mice (n = 5 per group) were injected i.p. with 100 µg of IgG1 isotype antibody (vehicle) or IL-17 neutralising antibodies (secukinumab, bimekizumab, MAB317 or Ab-IPL-IL-17™). In a selected time-point of 2 h, 24 h, 72 h, 7 days, 14 days and 21 days total IgG and IgG1 levels were determined by Elisa. The route, timing, and frequency of administration as well as the selected dosages of tested compounds were selected according to updated literature (24-28).

***Ex vivo* whole blood assay**

Whole blood culture and stimulation were performed as previously described with small modifications (29). Briefly, venous blood collected into lithium heparin tubes from patients with clinically diagnosed IBD (**Table 3**) was placed into 96-well plates with or without Ab-IPL-IL-17™ (10 µg/ml) at 37 °C, 5% CO_2 for 4 h. Following incubation, samples were centrifuged (300 g, 5 min at RT), and supernatants were collected and stored at −80 °C until IL-17A levels measurements by Elisa assay (30).

Elisa and Elisa Spot assays

The levels of IL-6 (DY406 and DY206, respectively, mouse and human kits) and TNF-α (DY210) in the *in vitro* and *ex vivo* supernatants were measured at 2 h or 24 h using commercially available enzyme-linked immunosorbent assay kits (Elisa kit, R&D System) according to the procedure previously described (25). Briefly, 100 µl of supernatants, diluted standards, quality controls, and dilution buffer (blank) were applied on a plate with the monoclonal antibody for 2 h. After washing, 100 µl of biotin-labelled antibody was added, and incubation continued for 1 h. The plate was washed, and 100 µl of the streptavidin–HRP conjugate was added, and the plate was incubated for a further 30 min period in the dark. The addition of 100 µl of the substrate and stop solution represented the last steps before the reading of absorbance (measured at 450 nm) on a microplate reader. Antigen levels in the samples were determined using a standard curve and expressed as pg/ml (31, 32). Similarly, serum level of IL-17A, IL-17F, total IgG and IgG1 (Elisa kit, R&D System) were measured at indicated time-points for *in vivo* neutralisation and immunogenicity assays.

Inflammatory exudates from the air pouch were incubated with the precoated proteome profiler array membranes according to the manufacturer's instructions (ARY006, R&D System). Dot plots were detected using the enhanced chemiluminescence detection kit and Image Quant 400 GE Healthcare software (GE Healthcare, Italy) and successively quantified using GS 800 imaging densitometer software (Bio-Rad) as extensively described (33, 34).

Haematological investigations

Standard laboratory procedures were used for blood sampling and measurements (34). Haematological investigations, for all experimental conditions, including blood count test, leukocyte, and sidereal formula were performed on citrated and not-anticoagulated blood samples, respectively. Serological tests were performed by CELL-DYN Sapphire purchased from Abbott S.R.L. (Milan, Italy). All procedures were conducted under strictly aseptic conditions.

Western blot analysis

Homogenates of cellular pellet (50 µg of protein) were subjected to SDS-PAGE (10 and 12% gel) using standard protocols, as previously described (31). The proteins were transferred to nitrocellulose membrane (0.2 µm nitrocellulose membrane, Trans-Blot®TurboTM, Transfer Pack, Bio-Rad) in transfer buffer (25 mM Tris-HCl pH 7.4 containing 192-mM glycine and 20% v/v methanol) at 400 mA for 2 h. Membranes were incubated for 2 h with non-fat dry milk (5% wt/v) in PBS supplemented with 0.1% (v/v) Tween 20 (PBS-T) at RT and then incubated with the following primary antibodies (1:1000) overnight at 4 °C before being washed three times with PBS-T: mouse monoclonal anti-beta-actin (MAB8929, R&D System), mouse monoclonal anti-NFκB (8242, Cell Signaling Technology, Milan, Italy), mouse monoclonal anti-IL-17RA (MAB448-SP, R&D System), mouse polyclonal anti-IL-17RC (AF2270-SP, R&D System), mouse monoclonal anti-Act-1 (sc-100647, Santa Cruz Biotechnology, Heidelberg, Germany). Blots were then incubated with a 1:3000 dilution of related HRP-conjugated secondary antibody (Dako, Copenhagen, Denmark) for 2 h at RT and finally washed three times with PBS-T. Protein bands were detected by using the enhanced chemiluminescence method (Clarity™ Western ECL Substrate, Bio-Rad) and Image Quant 400 GE Healthcare software (GE Healthcare). Bands were quantified using the GS 800 imaging densitometer software (Bio-Rad) and normalised with respective actin (25).

Flow Cytometry

HDBEC and hMDMs, collected after 24 h of treatment, were washed with PBS without Ca^{2+} and Mg^{2+} containing 25 mM lactose for 20 min at RT with occasional mixing. Cells were then incubated with FcR blocking agents (Miltenyi Biotec) in PBS without Ca^{2+} and Mg^{2+} containing lactose before staining cells with antibodies IL-17 RA (1:100) and IL-17 Receptor C (1:100). Moreover, HDBEC cells were also stained with ICAM-1 (1:50; clone HA58; Thermofisher scientific) and VCAM-1 (1:50;

Saviano A, *et al. Ann Rheum Dis* 2023; 82:1415–1428. doi: 10.1136/ard-2023-224479

clone 51-10C9; Thermofisher scientific). Protein expression was analysed by flow cytometry on BD LSRFortessa™ x-20 (BD, Biosciences, London, UK), and data were analysed using MRFlow and FlowJo software operation. The unspecific binding of antibodies was quantified by using corresponding isotype controls, gating strategy in **Fig. S12** (3, 35).

Sample processing for flow cytometry analysis

Mice were culled by cervical dislocation and lower limbs dissected and enzymatically digested as previously described (24). Briefly isolated joints were incubated in an enzyme cocktail consisting of 100μg/ml Collagenase D (Roche) and 100μg/ml DNase I (Sigma) diluted in RPMI containing 2% FBS at 37°C for 45 min. Undigested tissue was incubated with this enzyme cocktail again for an additional 30 min before cell suspension was passed through 100μM cell strainer (Vicedeal). Samples were washed in RPMI containing 2% FBS at 400g for 5 min. Cells were incubated in the RBC lysis buffer (Sigma) for 4 min at room temperature, washed at 400g for 5 min in PBS and resuspended in MACS buffer. All samples were blocked with FcR blocker (Biolegend) for 15 min prior to staining with the following of antibodies and Zombie Aqua (BV510) (all from Biolegend) for 20 minutes at 4°C prior to washing and fixation with 2% PFA: Anti-Cd11b BV650 (clone M1/70), anti-CD45 BV605 (clone: 109841), anti-Ly6C FITC (clone: 128005), anti-F4/80 APC-cy7 and anti-Ly6G APC (clone:IA8). Compensation controls were generated by killing cells under hot water for 1 minutes and adding zombie aqua to it for 15 minutes. After 15 minutes live spleen cells were added and run on fortessa. Samples were acquired using Fortessa-X20 and data analysed offline using FlowJo (V-10.2.6).

Elisa-based binding assay

Murine or Human IL-17RA-Fc (4481-MR or 177-IR, R&D System), and IL-17RC-Fc (2270-ML or 9284-MR, R&D System) were plated in 96 well plates at concentration of 2 µg/ml. Plates were incubated overnight at 4 ˚C, and then washed 3 times with TBST wash buffer and blocked for 1 h with 200 µl of protein-free T20 blocking buffer. Cytokines were biotinylated with EZ Link NHS-Biotin according to the manufacturer's instructions. Biotinylated cytokines and proteins (0-750 ng/ml) were incubated alone or in the presence of IL-17 neutralising antibodies (MAB421/MAB317 or Ab-IPL-IL-17™; 0-750 ng/ml) in wash buffer for 1 h. Plates were incubated for 30 min at RT before 3 washes with TBST and a 30 min incubation with Streptavidin-HRP. The colorimetric signal was developed with TMB, and the reaction stopped with sulfuric acid (stop solution). OD values were read on a SpectraMax M3 (Molecular Devices). The percentage of binding or inhibition was calculated as previously described (36).

Transwell migration

Chemotaxis was assessed using a transwell assay. IL-17 (10-500 ng/ml), nIL-17™ (10-500 ng/ml), formyl-methionyl-leucyl-phenylalanine (fMLP; 10^{-6} M, 59880-97-6, Sigma-Aldrich) alone or in combination with IL-17 neutralising antibodies (MAB317 or Ab-IPL-IL-17™, 10 µg/ml) were added to the bottom well of a Transwell-24 permeable support with 3.0 µm pores (Corning, NY, USA). Neutrophils ($2x10^5$ cells/well) were added to the upper chamber, which had a confluent HDBEC monolayer activated for 24 h with TNF-α (100 U/ml) and IFN-γ (10 ng/ml), and allowed to migrate for 2 h at 37 °C. Migrated neutrophils in the lower chamber were quantified using CountBright™ Absolute Counting Beads (Thermofisher scientific) by flow cytometry ($CD11b^+$ [1:100; clone ICRF44; BioLegend, Milan, Italy] / $CD16^+$ [1:100; clone 3G8; BioLegend, Milan, Italy] positive population) as previously described (35) (gating strategy in **Fig. S13**).

Human PBMC transendothelial migration assay

HDBEC seeded into 12-well plates were stimulated with TNF-α or IL-17 alone or in combination with IL-17 neutralising antibodies for 24 h, as previously described (35). Monolayers were washed with M199 containing 0.15% w/v BSA. PBMC (1 × 10^6) were allowed to adhere and migrate on stimulated HDBEC monolayers at 37 °C for 20 min. Non-adherent cells were removed by washing in M199 BSA, prior to the adherent cells being fixed with 2% glutaraldehyde (Sigma-Aldrich) for 15 min. Five random fields of view were imaged per well using phase-contrast microscopy (IX71; Olympus, Tokyo, Japan). Images were analysed offline using Image Pro 7 software (Media Cybernetics, Rockville, MD, USA), with adherent cells being defined as phase bright round cells, whilst transmigrated cells were shape changed and phase dark. PBMC adhesion was expressed as total number cells per mm^2. Transmigration was expressed as a percentage of adherent cells (11).

Static adhesion assay on PBL

HDBEC were seeded and stimulated with TNF-α (100 U ml^{-1}, R&D System) and/or IFN-γ (10 ng ml^{-1}, Peprotech) in presence of MIE extract (0.1-10 µg ml-1) or its corresponding vehicle (DMSO, 0.25%) for 4 h or 24 h before the assay with neutrophils or lymphocytes, respectively. PBL and neutrophils were isolated from healthy volunteers as described above prior to being added to activated HDBEC for 7 min at 37°C. Wells were washed twice with PBS to remove unbound cells and fixed with 2% glutaraldehyde for 10 min at RT. Glutaraldehyde was removed with several 1 ml washes of PBS (with Ca^{2+} and Mg^{2+}). Images were taken at five different fields in the centre of each well using phase-contrast microscopy (IX71; Olympus, Tokyo, Japan). Images were analysed offline using Image-pro 7.0 software (Media Cybernetics, Rockville, MD, USA), with adherent cells being defined as phase bright round cells, whilst transmigrated cells were shape changed and phase dark. PBL and neutrophils adhesion was expressed as the total number of cells per mm^2, while transmigration was expressed as a percentage of adherent cells (35).

Synthesis of a novel IL-17 neutralising antibody

Peptide synthesis and purification

Peptides were synthesized manually by the ultrasonic-assisted solid-phase peptide synthesis (US-SPPS), via the Fmoc/*t*Bu orthogonal protection strategy (37). In brief, Fmoc-deprotection (20% piperidine in DMF, 0.5 + 1 min treatments) and coupling (HBTU/HOBt as activating/additive agents, 5 min treatment) reactions were cyclically performed by ultrasonic irradiation to generate the resin-bound target peptide sequence. As solid supports, the 2-chlorotrityl chloride (2-CTC) (0.1 mmol from 1.70 mmol/g as loading substitution) or the Rink amide (0.1 mmol from 0.72 mmol/g as loading substitution) resins were employed depending on the C-terminal target as carboxylic acid or amide, respectively. Upon cleavage, crude peptides were purified by reverse-phase HPLC (RP-HPLC) using linear gradients of MeCN (0.1% TFA) in water (0.1% TFA), from 10 to 90% over 30 min, with a flow rate of 10 mL/min and UV detection at 220 nm. Final products were obtained by lyophilisation of the appropriate fractions after removal of the MeCN by rotary evaporation. All compounds examined for biological activity were of >97% purity, and the correct molecular ions were confirmed by mass spectrometry.

Circular dichroism

CD experiments were performed on a Jasco J-815 spectropolarimeter equipped with a PTC-423S/15 Peltier temperature controller. Cells with 0.1 cm path length and peptide concentration of 0.1 mM were used to record CD spectra between 200 and 260 nm, with a 1 nm bandwidth and a scan rate of 20 nm/min. Spectra were recorded at 20 °C. Spectra were signal-averaged over three scans, baseline-corrected by subtracting a buffer spectrum, and smoothed using the means-movement function. Spectra were analyzed for secondary structure composition using the BeStSel method (38).

Computational studies

nIL-17™ 3D structure prediction

PEP-FOLD 4 webserver (39-41) was used to predict the nIL-17™ structure from the amino acid sequence of the peptide. The primary sequence is submitted using the single letter code and the program predicts the 3D conformation by assembling predicted conformations of short local sequences using a greedy procedure driven by a coarse-grained energy score. PEP-FOLD 4 uses a new version of the force field (sOPEP2) that makes use of a Mie representation instead of the former Van der Waals representation for non-bonded interactions, and also includes an energy term using the Debye-Hueckel formalism to model pH, ionic strength dependence, and extremity blocking.

Molecular Docking

All docking calculations were conducted using the Schrödinger molecular modeling suite (version 2021-4). Protein structures were obtained from the Protein Data Bank (PDB). In particular, the structure of the complex of IL-17A (monomers A and B) with IL-17RA and IL-17RC receptors (PDB id: 7ZAN) (42) was selected to perform molecular docking studies with nIL-17™ peptide. The IL-17RA and IL-17RC receptors and nIL-17™ were prepared with the aid of the Protein Preparation Wizard panel of Maestro Suite (43) adding the missing hydrogen atoms and removing any water molecule with less than two hydrogen bonds to non-water molecules. In addition, the side chain ionization and tautomeric states were predicted, and the H-bonding network of the receptor refined minimizing the position of each hydrogen. Receptor grid generation tool of Glide software was used to generate the search grid around the IL-17A C-terminal region of monomers A and B to perform docking simulations of IL-17RA and IL-17RC, respectively (44-46). Then, docking calculations were performed using Glide 9.4 in its SP-peptide variant and employing the OPLS4 force field (44-46). Thus, the top-ranked complexes were selected and visually checked for a good chemical geometry.

Immunization and fusion protocols

The immunization protocol and the synthesis of a novel IL-17 neutralising antibody (Ab-IPL-IL-17™) were commissioned to ProteoGenix (France). All experimental details and procedures are reported in the Patent application (IT patent No. 102022000016722).

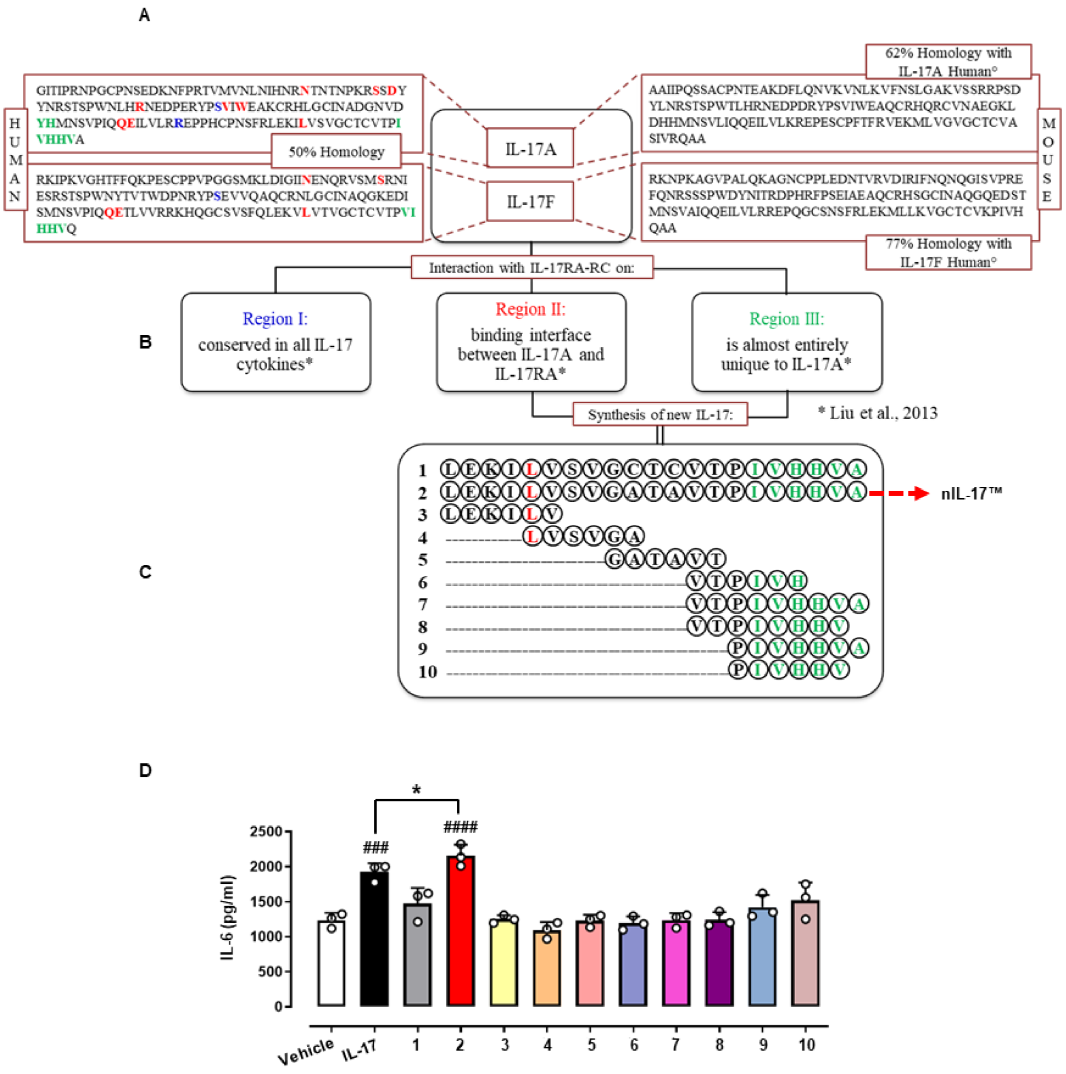

Fig. S1. Design peptide molecules that could be easily synthesized and used as stable surrogates of IL-17A/F. (**A**) The biological activity of both IL-17A and IL-17F are normally explicated due to the interaction of N- and C-terminal portions of these peptides on their own receptor/s. (**B**) Liu and coll. (47) identified specific regions of interactions between the cytokines of the IL-17 family and its receptor: regions 1 and 2, formed by the N-terminal region and central b-strands of IL-17A, respectively, that bind IL-17RA domain 1; and region 3, where the C-terminal region of IL-17A contacts the IL-17RA domain 2. (**B**) Region 1 is conserved in all IL-17 cytokines; region 2 has a major binding interface between IL-17A and IL-17RA thanks to a specific hydrophilic interaction; region 3 is almost entirely unique to IL-17A at this site. The higher or lesser affinity of IL-17RA for IL-17A and IL-17F is attributable to multiple interactions formed between these regions and the receptor (47). (**C**) Starting from these premises we aimed to design peptide molecules that could be easily synthesized and used as stable surrogates of IL-17A/F. (**D**) To assess the biological activity of synthesised peptides, IL-6 production was evaluated in NIH-3T3 cell supernatants following 24 h of incubation in presence of either IL-17A protein (50 ng/ml), and peptides from 1 to 10 (50 ng/ml). Data are presented as mean ± S.D. of n = 3 independent experiments. Statistical analysis was performed using the one-way ANOVA test followed by Bonferroni.$^{###}$P ≤ 0.001, $^{####}$P ≤ 0.0001 *vs* vehicle group; *P ≤ 0.05, *vs* IL-17 group.

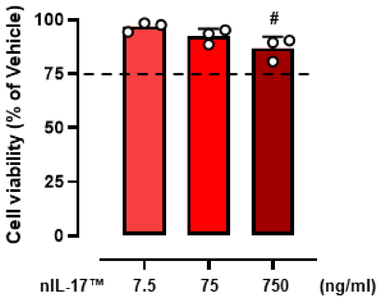

Fig. S2. *In vitro* cytotoxic examination for nIL-17™. *In vitro* cytotoxic examination, evaluated by MTT assay, for nIL-17™ was performed on murine embryonic fibroblast cell lines NIH-3T3, following 24 h of treatment with the selected concentrations (7.5-750 ng/ml). Dotted lines indicate 75 % of cell viability. Data are expressed as cell viability (% of vehicle) and presented as means ± S.D. of 3 independent experiments. Statistical analysis was conducted by one-way ANOVA followed by Bonferroni's for multiple comparisons. #P ≤ 0.05 *vs* vehicle group.

Saviano A, *et al. Ann Rheum Dis* 2023; 82:1415–1428. doi: 10.1136/ard-2023-224479

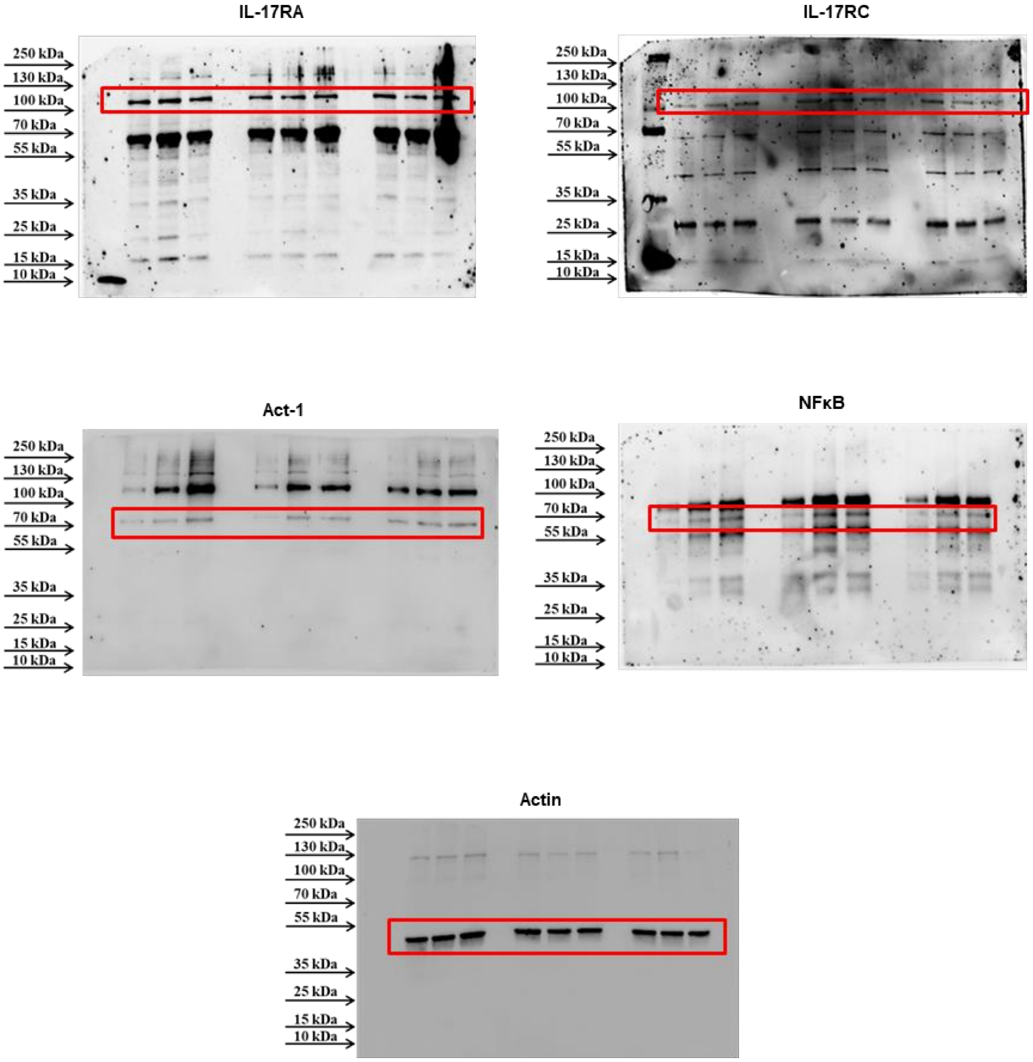

Fig. S3. Uncropped original western blots. Uncropped original western blots for IL-17RA (~120 kDa), IL-17RC (~110 kDa), Act-1 (~72 kDa), NFκB (~65 kDa) and Actin (~42 kDa) obtained from NIH-3T3 cells stimulated with IL-17 or nIL-17™ (50 ng/ml). Images represent three separate independent experiments run each with n = 3 per group pooled.

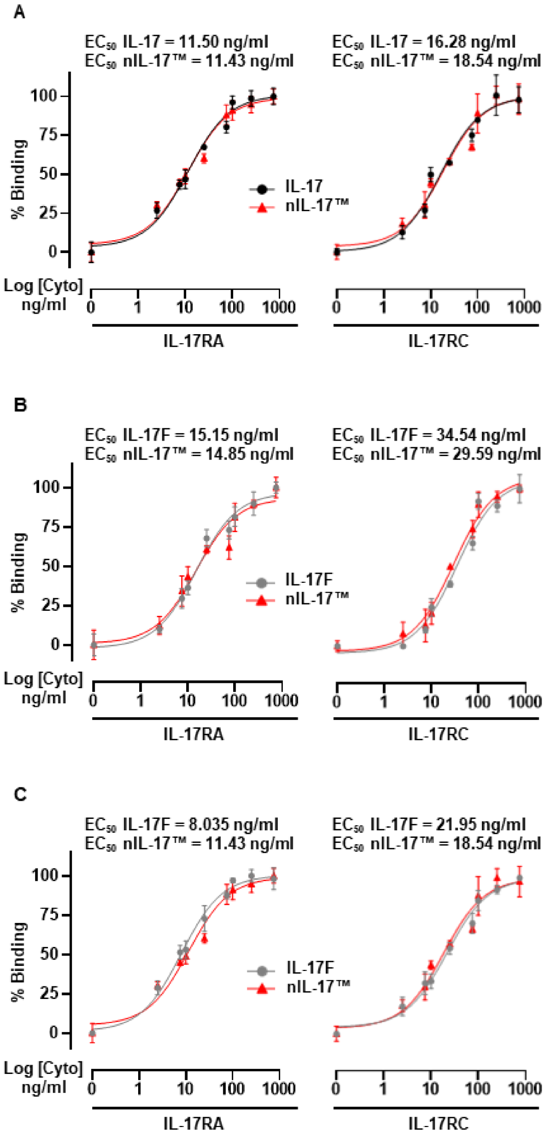

Fig. S4. Binding interaction of nIL-17™ with IL-17RA and RC. To evaluate the binding interaction of nIL-17™ with IL-17RA and RC, biotinylated (**A**) human IL-17A or both (**B**) mouse and (**C**) human IL-17F and nIL-17™ (0-750 ng/ml) were co-incubated for 30 min with IL-17RA-Fc or IL-17RC-Fc prior to fluorescence being measured. Data are presented as mean ± S.D. of n = 3 independent experiments.

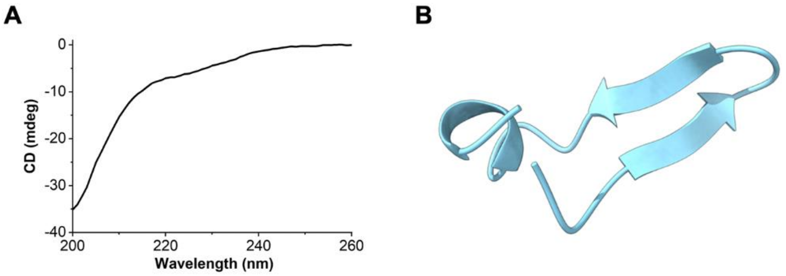

Fig. S5-I. (**A**) CD spectrum of nIL-17™ recorded in PBS buffer at 20 °C, and (**B**) best structural model of nIL-17™ generated by PEP-FOLD 4 software.

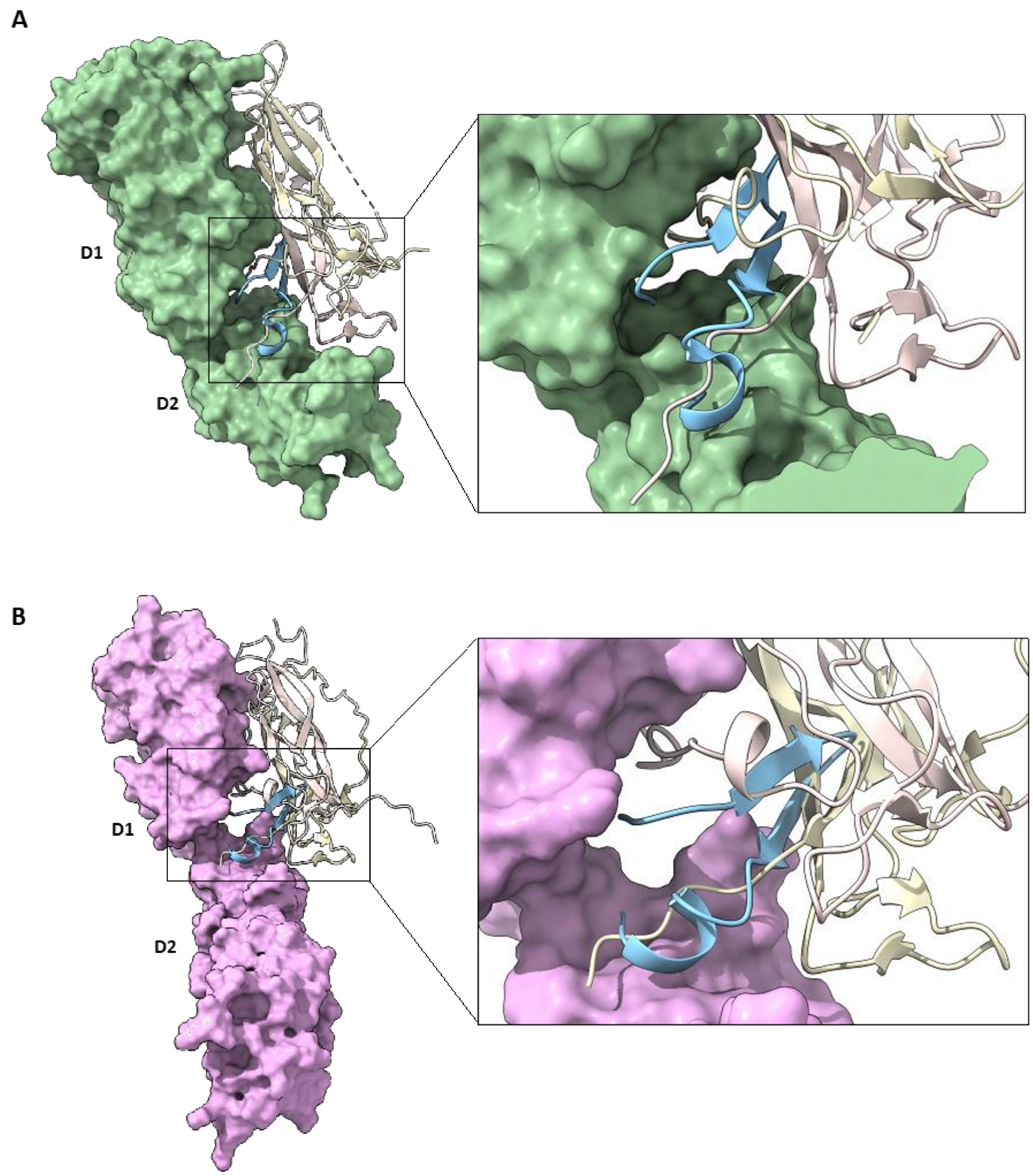

Fig. S5-II. Superposition of nIL-17™ (light blue) and IL-17A homodimer (protein monomers A and B are depicted as rose and light yellow ribbons, respectively) in complex with (**A**) IL-17RA (green surface), and (**B**) IL-17RC (violet surface).

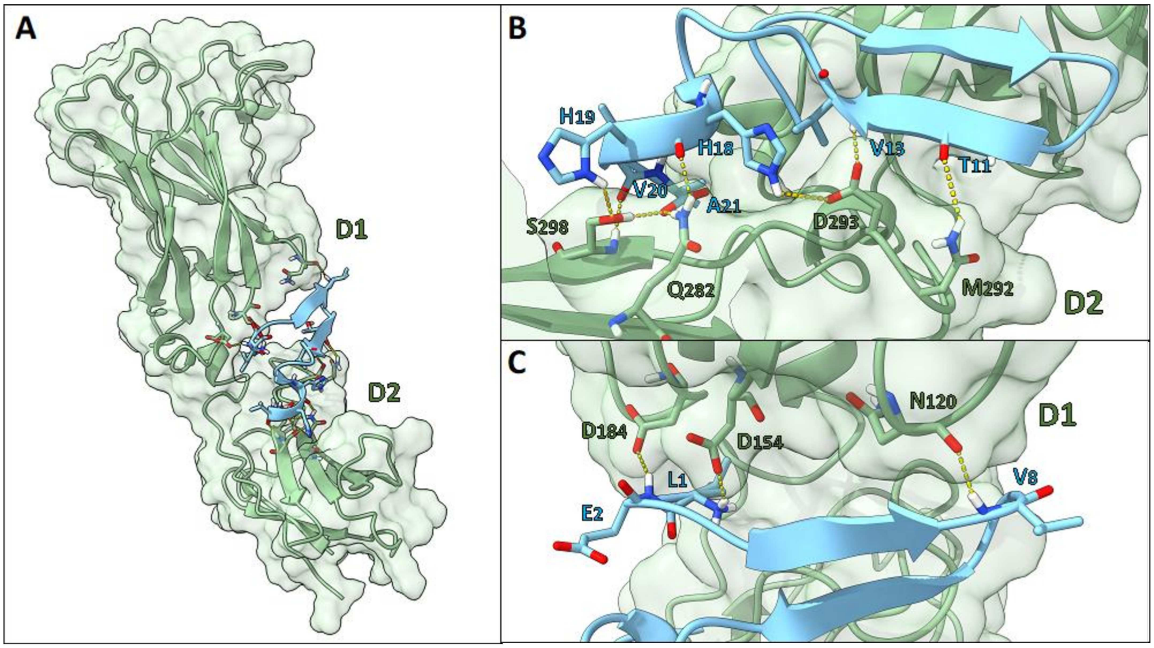

Fig. S5-III. (**A**) Docking-predicted binding mode of nIL-17™ (light blue) to IL-17RA receptor (green) (PDB id: 7ZAN). Zoom view of the binding mode of (**B**) the nIL-17™ C-terminal region with D2 domain, and (**C**) the nIL-17™ N-terminal region with D1 domain. Specifically, H18, H19, V20, and A21 of C-terminal region of nIL-17™ form H-bond interactions with S298 and Q282 of IL-17RA D2 domain. Moreover, V13 and T11 of nIL-17™ establish two H-bonds with D293 and N292 of the same receptor domain, respectively. Concerning the interactions between N-terminal region of nIL-17™ and IL-17RA D1 domain, the V8 amino acid of peptide β-turn is H-bonded to N120 of IL-17RA and, in addition, L1 of nIL-17™ N-terminal region can establish two H-bond interactions with D154 and D184 of D1 receptor domain.

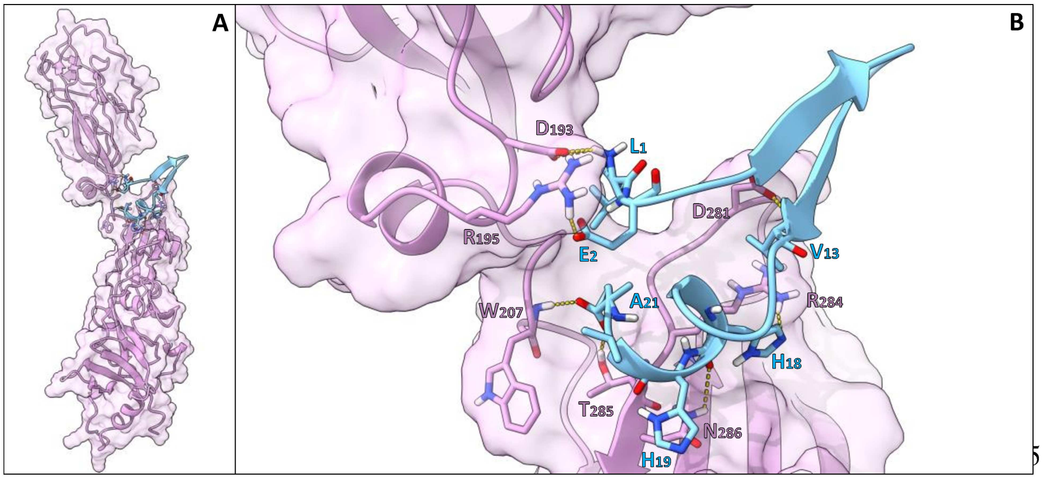

Fig. S5-IV. (**A**) Docking-predicted binding mode of nIL-17™ (light blue) to IL-17RC receptor (violet) (PDB id: 7ZAN), and (**B**) zoom view of the binding mode highlighting the H-bond interactions. Specifically, H18, H19, and A21 of C-terminal region of nIL-17™ form H-bond interactions with W207, R284, T285, and N286 of IL-17RC D2 domain. In addition, V13 of nIL-17™ forms a H-bond to D281 side chain of the same receptor domain, as in the case of IL-17RA. The interactions established by N-terminal region of nIL-17™, L1 and E2 can establish two H-bond interactions with D193 and R195 of the linker between D1 and D2 domains, respectively.

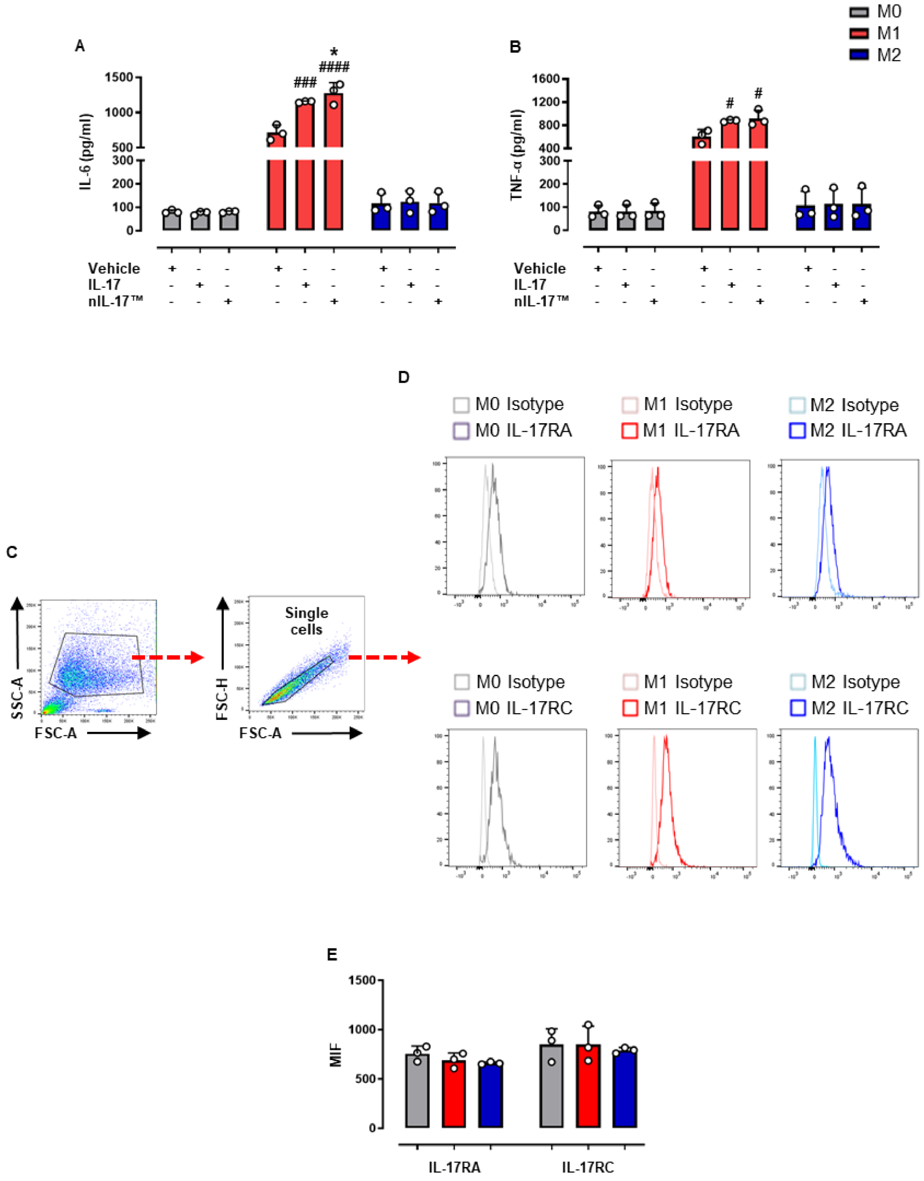

Fig. S6. Effect of nIL-17™ on M0, M1 and M2 macrophages. Macrophages, derived from primary human $CD14^+$ monocytes (M0) and stimulated with LPS and IFN-γ (M1) or IL-4 (M2) over 16 h, were treated with IL-17 vehicle, IL-17, or nIL-17™ (100 ng/ml) for 24 h. Supernatants from all experimental conditions were assayed by Elisa for (**A**) IL-6 and (**B**) TNF-α. Moreover, M0, M1 and M2 were gated (**C**) in their totality and singlet, before the identification of (**D**) IL-17Rs markers using flow cytometry analysis. (**E**) Histogram values indicate the total positive cells, in the different experimental conditions. Data are presented as means ± S.D. of n = 3 different healthy donors. Statistical analysis was conducted by one-way ANOVA followed by Bonferroni's for multiple comparisons. $^{\#}P \le 0.05$, $^{\#\#\#}P \le 0.001$, $^{\#\#\#\#}P \le 0.0001$ *vs* vehicle group; $^{*}P \le 0.05$ *vs* IL-17 group.

Saviano A, *et al. Ann Rheum Dis* 2023; 82:1415–1428. doi: 10.1136/ard-2023-224479

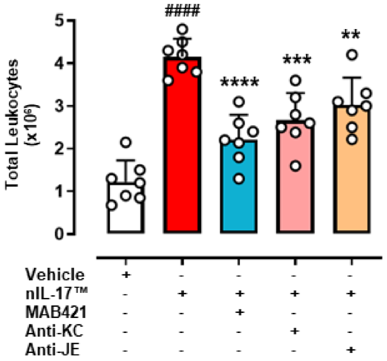

Fig. S7. Effect of MAB421, anti-KC or anti-JE on nIL-17™-induced *in vivo* inflammation. Mice were treated with IL-17 vehicle (0.5% CMC), nIL-17™ (1 µg/pouch) alone or in co-administration with MAB421, anti-KC or anti-JE (10 µg/pouch), and thereafter total CD45^{+} leukocyte numbers were quantified. Values are presented as means ± S.D. of n = 7 mice per group. Statistical analysis was conducted by one-way ANOVA followed by Bonferroni's for multiple comparisons. $^{####}$P ≤ 0.0001 *vs* vehicle group; **P ≤ 0.01, ***P ≤ 0.001, ****P ≤ 0.0001 *vs* nIL-17™ group.

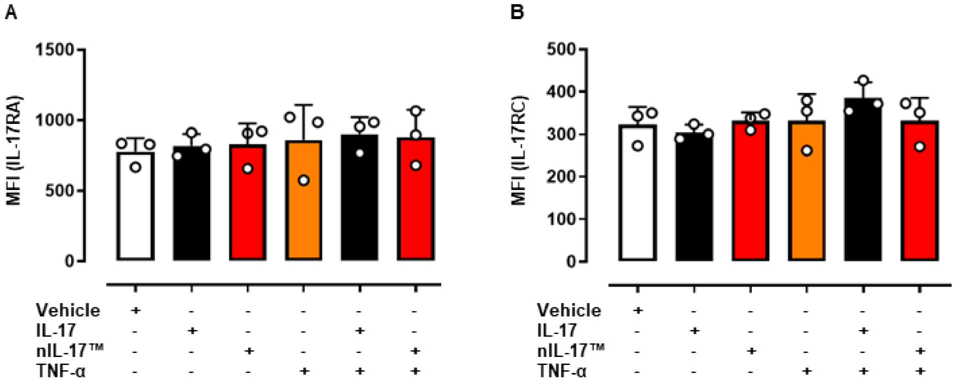

Fig. S8. Expression of IL-17RA and RC on HDBEC. HDBEC were treated with IL-17 vehicle (HCl 4 mM PBS), IL-17 (100 ng/ml) or nIL-17™ (100 ng/ml), alone or in combination with TNF-α (100 U/ml) for 24 h. Cells were washed, gated in their totality and singlet before the identification of IL-17Rs. Histogram values indicate the total positive cells, in the different experimental conditions, of (**A**) IL-17RA and (**B**) IL-17RC. Data were presented as means ± S.D. of n = 3 healthy donors. Statistical analysis was conducted by one-way ANOVA followed by Bonferroni's for multiple comparisons.

Saviano A, *et al. Ann Rheum Dis* 2023; 82:1415–1428. doi: 10.1136/ard-2023-224479

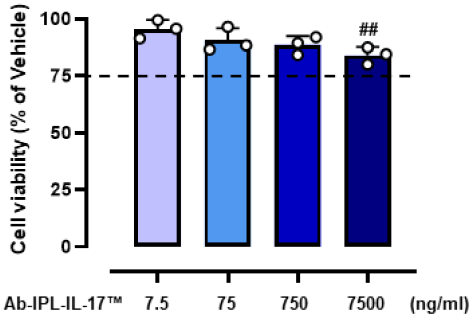

Fig. S9. *In vitro* cytotoxic examination for Ab-IPL-IL-17™. *In vitro* cytotoxic examination, evaluated by MTT assay, for Ab-IPL-IL-17™ was performed on murine embryonic fibroblast cell lines NIH-3T3, following 24 h of treatment with the selected concentrations (7.5-7500 ng/ml). Dotted lines indicate 75 % of cell viability. Data are expressed as cell viability (% of vehicle) and presented as means ± S.D. of 3 independent experiments. Statistical analysis was conducted by one-way ANOVA followed by Bonferroni's for multiple comparisons. $^{\#\#}P \leq 0.01$ *vs* vehicle group.

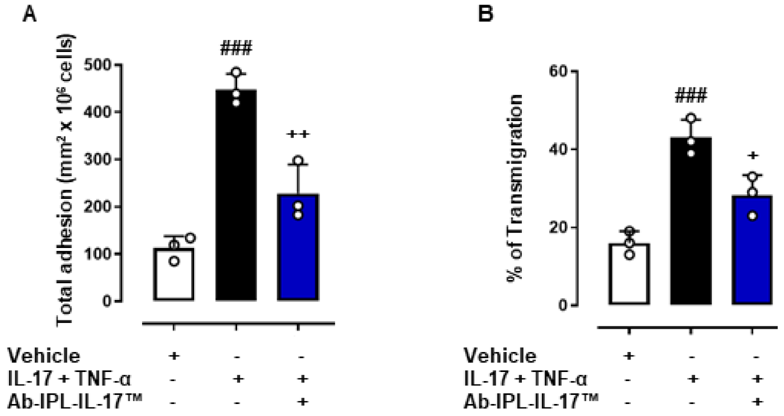

Fig. S10. Ab-IPL-IL-17™ reduced PBL adhesion and transmigration. HDBECs were treated with IL-17 vehicle (HCl 4 mM PBS), IL-17 (100 ng/ml) plus TNF-α (100 U/ml), alone or in combination with Ab-IPL-IL-17™ (10 µg/ml) for 24 h. Phase bright PBLs were considered (**A**) adherent, whereas phase-dark were quantified as (**B**) transmigrated (% of adherent cells). Data are presented as means ± S.D. of n = 3 independent healthy donors. Statistical analysis was conducted by one-way ANOVA followed by Bonferroni's for multiple comparisons. $^{\#\#\#}P \leq 0.001$ *vs* vehicle group; $^{+}P \leq 0.05$, $^{++}P \leq 0.01$ *vs* IL-17 + TNF-α group.

Saviano A, *et al. Ann Rheum Dis* 2023; 82:1415–1428. doi: 10.1136/ard-2023-224479

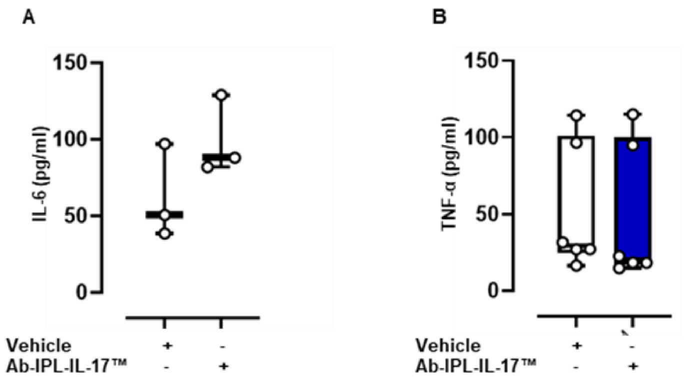

Fig. S11. Human whole blood, collected from IBD patients, treated with Ab-IPL-IL-17™. Human whole blood, collected from IBD patients, was treated with or without Ab-IPL-IL-17™ (10 µg/ml) for 4 h (37 °C, 5% CO_2). Serum was collected for the assessment of (**A**) IL-6 and (**B**) TNF-α by Elisa assay. Data are median ± interquantile ranges (min 25%, max 75%) for n = 3 for IL-6 and n = 6 independent donors for TNF-α detection. Statistical analysis was conducted by one-way ANOVA followed by Bonferroni's for multiple comparisons.

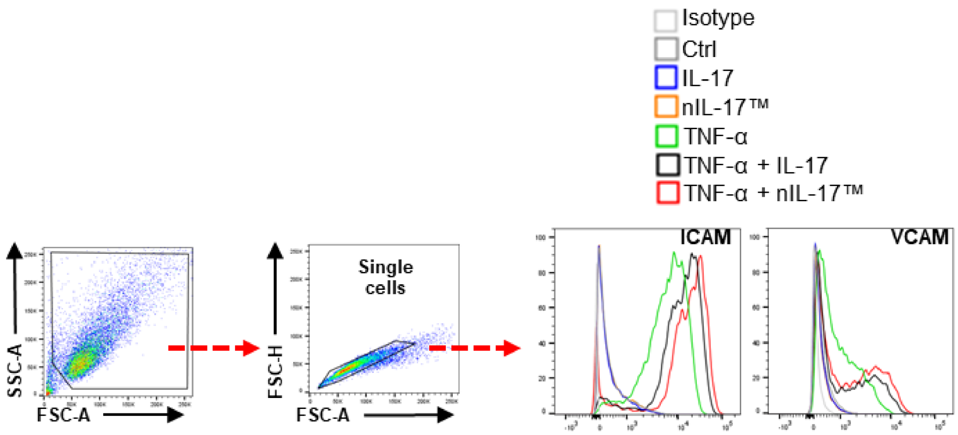

Fig. S12. Identification of ICAM-1 and VCAM-1 expression on HDBEC. HDBEC were treated with IL-17 vehicle (HCl 4 mM PBS), IL-17 (100 ng/ml) or nIL-17™ (100 ng/ml), alone or in combination with TNF-α (100 U/ml) for 24 h. Cells were washed, stained and flow cytometry was performed. Singlets were gated on and ICAM-1 and VCAM-1 expression was measured. Related isotype controls were included for all antibodies used.

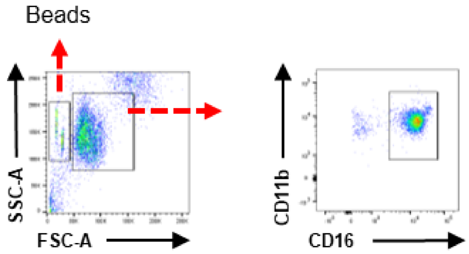

Fig. S13. Chemotaxis assay on neutrophils. For the chemotaxis assay, neutrophils collected from the bottom of the transwell plate were analsysed by flow cytometry. Total neutrophils were gated on based on FSC/SSC and markers CD11b⁺/CD16⁺. Counting beads were also spiked into samples to obtain absolute cell counts.

	α-helix (%)	Antiparallel β-sheet (%)	Parallel β-sheet (%)	β-turn (%)	Other (%)
nIL-17™	4.3	24.4	0.3	18.9	52.1

Auxiliary Table 1. Secondary structure content estimation of nIL-17™ derived from the circular dichroism spectrum at 20 °C.

Saviano A, *et al. Ann Rheum Dis* 2023; 82:1415–1428. doi: 10.1136/ard-2023-224479

```
sp|Q8NAC3|I17RC_HUMAN       MPVPWFLLSLALGRSPVVLSLERLVGPQDATHCSPVSLEPWGDEERLRVQFLAQQSLSLA    60
sp|Q8NAC3-2|I17RC_HUMAN     MPVPWFLLSLALGRSPVVLSLERLVGPQDATHCSP-------------------------    35
                            ***********************************

sp|Q8NAC3|I17RC_HUMAN       PVTAATARTALSGLSGADGRREERGRGKSWVCLSLGGSGNTEPQKKGLSCRLWDSDILCL    120
sp|Q8NAC3-2|I17RC_HUMAN     ----------------------------------------------GLSCRLWDSDILCL    49
                                                                          **************

sp|Q8NAC3|I17RC_HUMAN       PGDIVPAPGPVLAPTHLQTELVLRCQKETDCDLCLRVAVHLAVHGHWEEPEDEEKFGGAA    180
sp|Q8NAC3-2|I17RC_HUMAN     PGDIVPAPGPVLAPTHLQTELVLRCQKETDCDLCLRVAVHLAVHGHWEEPEDEEKFGGAA    109
                            ************************************************************

sp|Q8NAC3|I17RC_HUMAN       DSGVEEPRNASLQAQVVLSFQAYPTARCVLLEVQVPAALVQFGQSVGSVVYDCFEAALGS    240
sp|Q8NAC3-2|I17RC_HUMAN     DSGVEEPRNASLQAQVVLSFQAYPTARCVLLEVQVPAALVQFGQSVGSVVYDCFEAALGS    169
                            ************************************************************

sp|Q8NAC3|I17RC_HUMAN       EVRIWSYTQPRYEKELNHTQQLPDCRGLEVWNSIPSCWALPWLNVSADGDNVHLVLNVSE    300
sp|Q8NAC3-2|I17RC_HUMAN     EVRIWSYTQPRYEKELNHTQQLPDCRGLEVWNSIPSCWALPWLNVSADGDNVHLVLNVSE    229
                            ************************************************************

sp|Q8NAC3|I17RC_HUMAN       EQHFGLSLYWNQVQGPPKPRWHKNLTGPQIITLNHTDLVPCLCIQVWPLEPDSVRTNICP    360
sp|Q8NAC3-2|I17RC_HUMAN     EQHFGLSLYWNQVQGPPKPRWHKNLTGPQIITLNHTDLVPCLCIQVWPLEPDSVRTNICP    289
                            ************************************************************

sp|Q8NAC3|I17RC_HUMAN       FREDPRAHQNLWQAARLQLLTLQSWLLDAPCSLPAEAALCWRAPGGDPCQPLVPPLSWEN    420
sp|Q8NAC3-2|I17RC_HUMAN     FREDPRAHQNLWQAARLQLLTLQSWLLDAPCSLPAEAALCWRAPGGDPCQPLVPPLSWEN    349
                            ************************************************************

sp|Q8NAC3|I17RC_HUMAN       VTVDKVLEFPLLKGHPNLCVQVNSSEKLQLQECLWADSLGPLKDDVLLLETRGPQDNRSL    480
sp|Q8NAC3-2|I17RC_HUMAN     VTVDKVLEFPLLKGHPNLCVQVNSSEKLQLQECLWADSLGPLKDDVLLLETRGPQDNRSL    409
                            ************************************************************

sp|Q8NAC3|I17RC_HUMAN       CALEPSGCTSLPSKASTRAARLGEYLLQDLQSGQCLQLWDDDLGALWACPMDKYIHKRWA    540
sp|Q8NAC3-2|I17RC_HUMAN     CALEPSGCTSLPSKASTRAARLGEYLLQDLQSGQCLQLWDDDLGALWACPMDKYIHKRWA    469
                            ************************************************************

sp|Q8NAC3|I17RC_HUMAN       LVWLACLLFAAALSLILLLKKDHAKGWLRLLKQDVRSGAAARGRAALLLYSADDSGFERL    600
sp|Q8NAC3-2|I17RC_HUMAN     LVWLACLLFAAALSLILLLKKDHAKGWLRLLKQDVRSGAAARGRAALLLYSADDSGFERL    529
                            ************************************************************

sp|Q8NAC3|I17RC_HUMAN       VGALASALCQLPLRVAVDLWSRRELSAQGPVAWFHAQRRQTLQEGGVVVLLFSPGAVALC    660
sp|Q8NAC3-2|I17RC_HUMAN     VGALASALCQLPLRVAVDLWSRRELSAQGPVAWFHAQRRQTLQEGGVVVLLFSPGAVALC    589
                            ************************************************************

sp|Q8NAC3|I17RC_HUMAN       SEWLQDGVSGPGAHGPHDAFRASLSCVLPDFLQGRAPGSYVGACFDRLLHPDAVPALFRT    720
sp|Q8NAC3-2|I17RC_HUMAN     SEWLQDGVSGPGAHGPHDAFRASLSCVLPDFLQGRAPGSYVGACFDRLLHPDAVPALFRT    649
                            ************************************************************

sp|Q8NAC3|I17RC_HUMAN       VPVFTLPSQLPDFLGALQQPRAPRSGRLQERAEQVSRALQPALDSYFHPPGTPAPGRGVG    780
sp|Q8NAC3-2|I17RC_HUMAN     VPVFTLPSQLPDFLGALQQPRAPRSGRLQERAEQVSRALQPALDSYFHPPGTPAPGRGVG    709
                            ************************************************************

sp|Q8NAC3|I17RC_HUMAN       PGAGPGAGDGT        791
sp|Q8NAC3-2|I17RC_HUMAN     PGAGPGAGDGT        720
                            ***********
```

Auxiliary Table 2. Sequence alignment of isoform 1 (Q8NAC3) and isoform 2 (Q8NAC3-2) of IL-17RC.

Saviano A, *et al. Ann Rheum Dis* 2023; 82:1415–1428. doi: 10.1136/ard-2023-224479

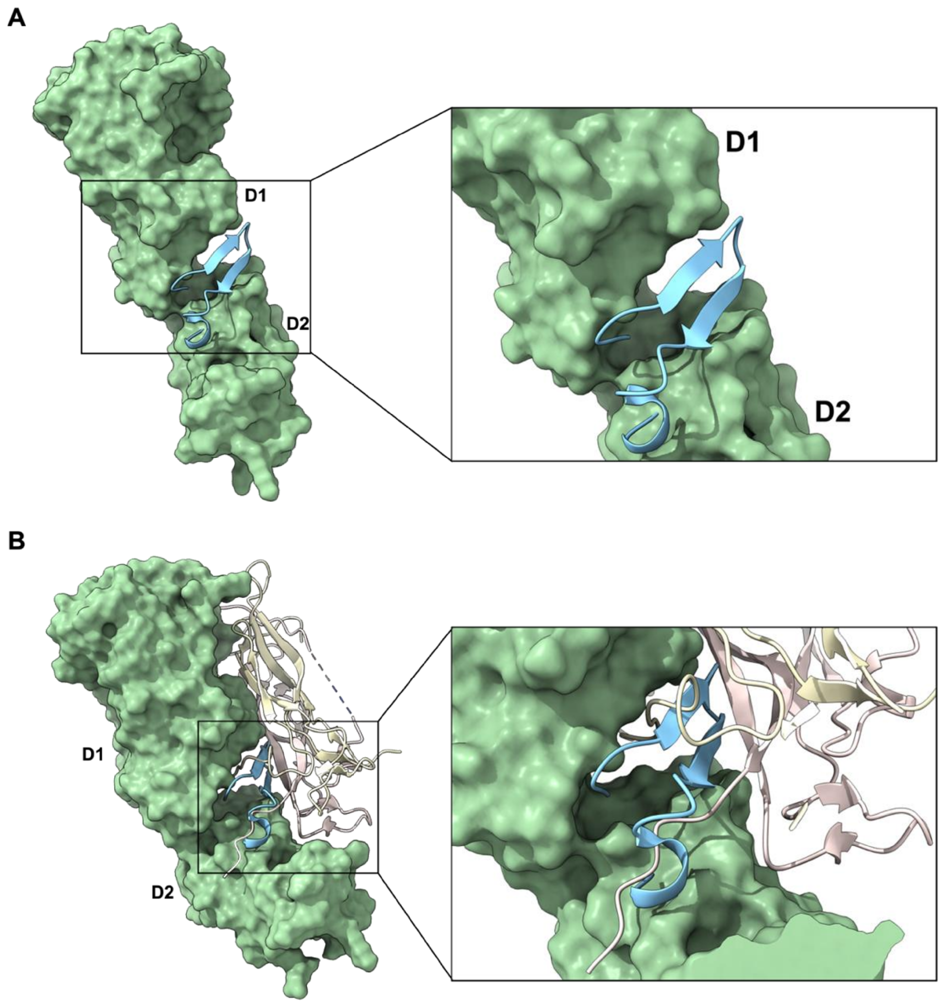

Auxiliary Fig. 1. (**A**) Docking-predicted binding mode of nIL-17™ to IL-17RA receptor (PDB id: 7ZAN). IL-17RA and nIL-17™ are depicted as green surface and light blue ribbon, respectively. (**B**) Superposition of nIL-17™ and IL-17A homodimer (protein monomers A and B are depicted as rose and light yellow ribbons, respectively) in complex with IL-17RA receptor.

Saviano A, *et al. Ann Rheum Dis* 2023; 82:1415–1428. doi: 10.1136/ard-2023-224479

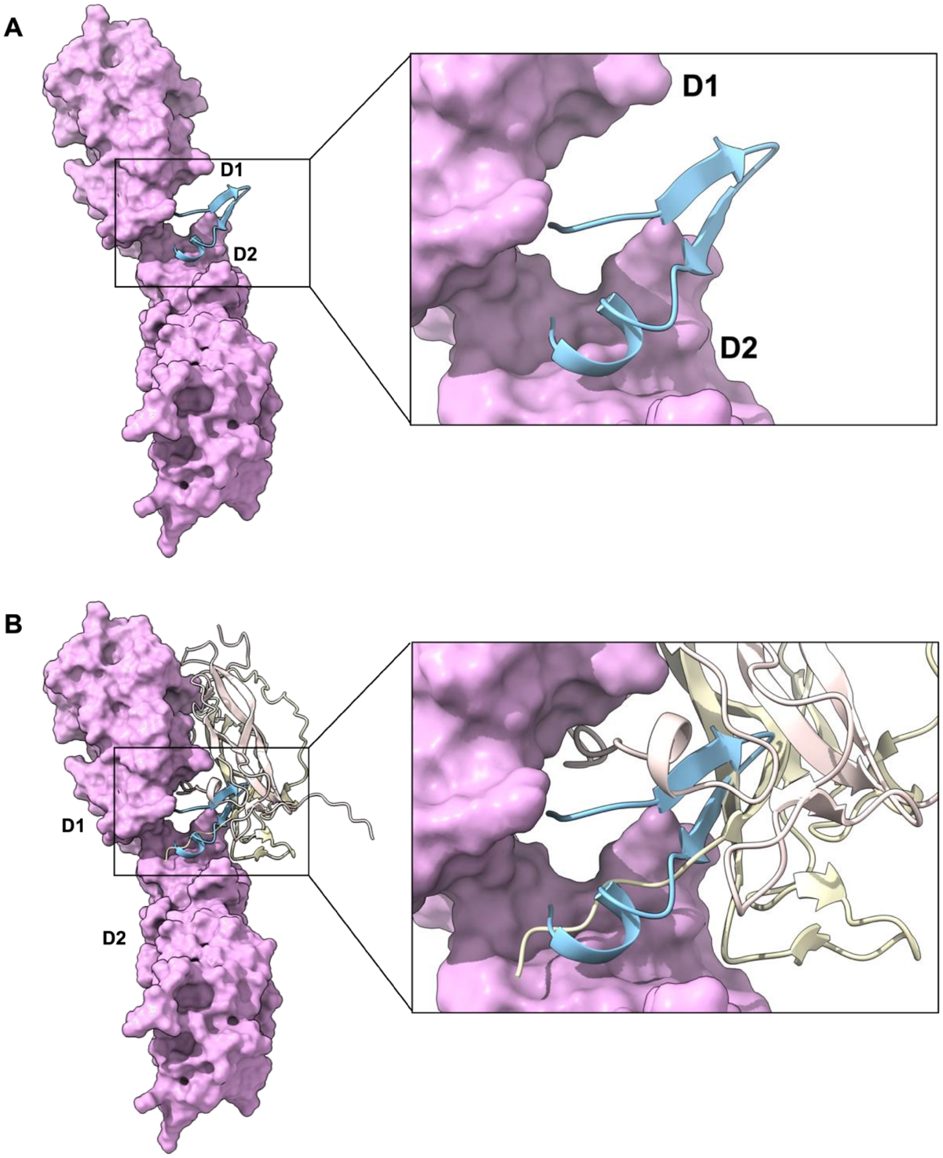

Auxiliary Fig. 2. (**A**) Docking-predicted binding mode of nIL-17™ to IL-17RC receptor (PDB id: 7ZAN). IL-17RC and nIL-17™ are depicted as violet surface and light blue ribbon, respectively. (**B**) Superposition of nIL-17™ and IL-17A homodimer (protein monomers A and B are depicted as rose and light yellow ribbons, respectively) in complex with IL-17RC receptor.

SI References

1. Mangan PR, Su LJ, Jenny V*, et al.* Dual Inhibition of Interleukin-23 and Interleukin-17 Offers Superior Efficacy in Mouse Models of Autoimmunity. *J Pharmacol Exp Ther* 2015;**354**(2):152-65. doi:10.1124/jpet.115.224246

2. Saviano A, Raucci F, Casillo GM*, et al.* Anti-inflammatory and immunomodulatory activity of Mangifera indica L. reveals the modulation of COX-2/mPGES-1 axis and Th17/Treg ratio. *Pharmacol Res* 2022;**182**:106283. doi:10.1016/j.phrs.2022.106283

3. Krautter F, Recio C, Hussain MT*, et al.* Characterisation of endogenous Galectin-1 and -9 expression in monocyte and macrophage subsets under resting and inflammatory conditions. *Biomed Pharmacother* 2020;**130**:110595. doi:10.1016/j.biopha.2020.110595

4. Martinez FO, Gordon S, Locati M*, et al.* Transcriptional profiling of the human monocyte-to-macrophage differentiation and polarization: new molecules and patterns of gene expression. *J Immunol* 2006;**177**(10):7303-11. doi:10.4049/jimmunol.177.10.7303

5. Jovanovic DV, Di Battista JA, Martel-Pelletier J*, et al.* Modulation of TIMP-1 synthesis by antiinflammatory cytokines and prostaglandin E2 in interleukin 17 stimulated human monocytes/macrophages. *J Rheumatol* 2001;**28**(4):712-8.

6. Kapellos TS, Taylor L, Feuerborn A*, et al.* Cannabinoid receptor 2 deficiency exacerbates inflammation and neutrophil recruitment. *Faseb j* 2019;**33**(5):6154-67. doi:10.1096/fj.201802524R

7. Hot A, Lenief V, Miossec P. Combination of IL-17 and TNFα induces a pro-inflammatory, pro-coagulant and pro-thrombotic phenotype in human endothelial cells. *Ann Rheum Dis* 2012;**71**(5):768-76. doi:10.1136/annrheumdis-2011-200468

8. Riedhammer C, Halbritter D, Weissert R. Peripheral Blood Mononuclear Cells: Isolation, Freezing, Thawing, and Culture. *Methods Mol Biol* 2016;**1304**:53-61. doi:10.1007/7651_2014_99

9. McGettrick HM, Hunter K, Moss PA*, et al.* Direct observations of the kinetics of migrating T cells suggest active retention by endothelial cells with continual bidirectional migration. *J Leukoc Biol* 2009;**85**(1):98-107. doi:10.1189/jlb.0508301

10. Ahmed SR, McGettrick HM, Yates CM*, et al.* Prostaglandin D2 regulates CD4+ memory T cell trafficking across blood vascular endothelium and primes these cells for clearance across lymphatic endothelium. *J Immunol* 2011;**187**(3):1432-9. doi:10.4049/jimmunol.1100299

11. McGettrick HM, Butler LM, Nash GB. Analysis of leukocyte migration through monolayers of cultured endothelial cells. *Methods Mol Biol* 2007;**370**:37-54. doi:10.1007/978-1-59745-353-0_4

12. Tull SP, Yates CM, Maskrey BH*, et al.* Omega-3 Fatty acids and inflammation: novel interactions reveal a new step in neutrophil recruitment. *PLoS Biol* 2009;**7**(8):e1000177. doi:10.1371/journal.pbio.1000177

13. Iqbal AJ, Krautter F, Blacksell IA*, et al.* Galectin-9 mediates neutrophil capture and adhesion in a CD44 and β2 integrin-dependent manner. *Faseb j* 2022;**36**(1):e22065. doi:10.1096/fj.202100832R

14. Kelly S, Humby F, Filer A*, et al.* Ultrasound-guided synovial biopsy: a safe, well-tolerated and reliable technique for obtaining high-quality synovial tissue from both large and small joints in early arthritis patients. *Ann Rheum Dis* 2015;**74**(3):611-7. doi:10.1136/annrheumdis-2013-204603

15. Filer A, de Pablo P, Allen G*, et al.* Utility of ultrasound joint counts in the prediction of rheumatoid arthritis in patients with very early synovitis. *Ann Rheum Dis* 2011;**70**(3):500-7. doi:10.1136/ard.2010.131573
16. Young SP, Kapoor SR, Viant MR*, et al.* The impact of inflammation on metabolomic profiles in patients with arthritis. *Arthritis Rheum* 2013;**65**(8):2015-23. doi:10.1002/art.38021
17. Aletaha D, Neogi T, Silman AJ*, et al.* 2010 Rheumatoid arthritis classification criteria: an American College of Rheumatology/European League Against Rheumatism collaborative initiative. *Arthritis Rheum* 2010;**62**(9):2569-81. doi:10.1002/art.27584
18. McGettrick HM, Smith E, Filer A*, et al.* Fibroblasts from different sites may promote or inhibit recruitment of flowing lymphocytes by endothelial cells. *Eur J Immunol* 2009;**39**(1):113-25. doi:10.1002/eji.200838232
19. Salmon M, Scheel-Toellner D, Huissoon AP*, et al.* Inhibition of T cell apoptosis in the rheumatoid synovium. *J Clin Invest* 1997;**99**(3):439-46. doi:10.1172/jci119178
20. Parsonage G, Filer A, Bik M*, et al.* Prolonged, granulocyte-macrophage colony-stimulating factor-dependent, neutrophil survival following rheumatoid synovial fibroblast activation by IL-17 and TNFalpha. *Arthritis Res Ther* 2008;**10**(2):R47. doi:10.1186/ar2406
21. Dallenbach K, Maurer P, Röhn T*, et al.* Protective effect of a germline, IL-17-neutralizing antibody in murine models of autoimmune inflammatory disease. *Eur J Immunol* 2015;**45**(4):1238-47. doi:10.1002/eji.201445017
22. Liu L, Lu J, Allan BW*, et al.* Generation and characterization of ixekizumab, a humanized monoclonal antibody that neutralizes interleukin-17A. *J Inflamm Res* 2016;**9**:39-50. doi:10.2147/jir.S100940
23. Wu M, Zhang H, Li Q*, et al.* Pharmacokinetics, Pharmacodynamics, Safety, Tolerability, and Immunogenicity of the QX002N anti-IL-17 Monoclonal Antibody: A Phase I, Randomized, Double-Blind, Single Ascending Dose Study in Healthy Chinese Volunteers. *Front Pharmacol* 2021;**12**:794054. doi:10.3389/fphar.2021.794054
24. Raucci F, Iqbal AJ, Saviano A*, et al.* IL-17A neutralizing antibody regulates monosodium urate crystal-induced gouty inflammation. *Pharmacol Res* 2019;**147**:104351. doi:10.1016/j.phrs.2019.104351
25. Raucci F, Saviano A, Casillo GM*, et al.* IL-17-induced inflammation modulates the mPGES-1/PPAR-γ pathway in monocytes/macrophages. *Br J Pharmacol* 2022;**179**(9):1857-73. doi:10.1111/bph.15413
26. McInnes IB, Asahina A, Coates LC*, et al.* Bimekizumab in patients with psoriatic arthritis, naive to biologic treatment: a randomised, double-blind, placebo-controlled, phase 3 trial (BE OPTIMAL). *Lancet* 2023;**401**(10370):25-37. doi:10.1016/s0140-6736(22)02302-9
27. Ma H, Zhang W, Liu K*, et al.* Generation and characterization of QLS22001, a humanized monoclonal antibody that neutralizes IL-17A and IL-17F with an extended half-life. *Int Immunopharmacol* 2023;**117**:109947. doi:10.1016/j.intimp.2023.109947
28. Simopoulou T, Tsiogkas SG, Zafiriou E*, et al.* Secukinumab, ixekizumab, bimekizumab and brodalumab for psoriasis and psoriatic arthritis. *Drugs Today (Barc)* 2023;**59**(3):135-67. doi:10.1358/dot.2023.59.3.3419557

29. Papandreou V, Kavrochorianou N, Katsoulas T*, et al.* Adrenergic Effect on Cytokine Release After Ex Vivo Healthy Volunteers' Whole Blood LPS Stimulation. *Inflammation* 2016;**39**(3):1069-75. doi:10.1007/s10753-016-0338-y
30. Myrianthefs P, Karatzas S, Venetsanou K*, et al.* Seasonal variation in whole blood cytokine production after LPS stimulation in normal individuals. *Cytokine* 2003;**24**(6):286-92. doi:10.1016/j.cyto.2003.08.005
31. Maione F, Iqbal AJ, Raucci F*, et al.* Repetitive Exposure of IL-17 Into the Murine Air Pouch Favors the Recruitment of Inflammatory Monocytes and the Release of IL-16 and TREM-1 in the Inflammatory Fluids. *Front Immunol* 2018;**9**:2752. doi:10.3389/fimmu.2018.02752
32. Baradaran Rahimi V, Rakhshandeh H, Raucci F*, et al.* Anti-Inflammatory and Anti-Oxidant Activity of Portulaca oleracea Extract on LPS-Induced Rat Lung Injury. *Molecules* 2019;**24**(1). doi:10.3390/molecules24010139
33. Cristiano C, Volpicelli F, Lippiello P*, et al.* Neutralization of IL-17 rescues amyloid-β-induced neuroinflammation and memory impairment. *Br J Pharmacol* 2019;**176**(18):3544-57. doi:10.1111/bph.14586
34. Saviano A, Casillo GM, Raucci F*, et al.* Supplementation with ribonucleotide-based ingredient (Ribodiet®) lessens oxidative stress, brain inflammation, and amyloid pathology in a murine model of Alzheimer. *Biomed Pharmacother* 2021;**139**:111579. doi:10.1016/j.biopha.2021.111579
35. Mansour AA, Raucci F, Sevim M*, et al.* Galectin-9 supports primary T cell transendothelial migration in a glycan and integrin dependent manner. *Biomed Pharmacother* 2022;**151**:113171. doi:10.1016/j.biopha.2022.113171
36. Ting JP, Tung F, Antonysamy S*, et al.* Utilization of peptide phage display to investigate hotspots on IL-17A and what it means for drug discovery. *PLoS One* 2018;**13**(1):e0190850. doi:10.1371/journal.pone.0190850
37. Merlino F, Tomassi S, Yousif AM*, et al.* Boosting Fmoc Solid-Phase Peptide Synthesis by Ultrasonication. *Org Lett* 2019;**21**(16):6378-82. doi:10.1021/acs.orglett.9b02283
38. Micsonai A, Wien F, Kernya L*, et al.* Accurate secondary structure prediction and fold recognition for circular dichroism spectroscopy. *Proc Natl Acad Sci U S A* 2015;**112**(24):E3095-103. doi:10.1073/pnas.1500851112
39. Lamiable A, Thévenet P, Rey J*, et al.* PEP-FOLD3: faster de novo structure prediction for linear peptides in solution and in complex. *Nucleic Acids Res* 2016;**44**(W1):W449-54. doi:10.1093/nar/gkw329
40. Tufféry P, Derreumaux P. A refined pH-dependent coarse-grained model for peptide structure prediction in aqueous solution. *Front Bioinform* 2023;**3**:1113928. doi:10.3389/fbinf.2023.1113928
41. Binette V, Mousseau N, Tuffery P. A Generalized Attraction-Repulsion Potential and Revisited Fragment Library Improves PEP-FOLD Peptide Structure Prediction. *J Chem Theory Comput* 2022;**18**(4):2720-36. doi:10.1021/acs.jctc.1c01293
42. Goepfert A, Barske C, Lehmann S*, et al.* IL-17-induced dimerization of IL-17RA drives the formation of the IL-17 signalosome to potentiate signaling. *Cell Rep* 2022;**41**(3):111489. doi:10.1016/j.celrep.2022.111489

43. Sastry GM, Adzhigirey M, Day T*, et al.* Protein and ligand preparation: parameters, protocols, and influence on virtual screening enrichments. *J Comput Aided Mol Des* 2013;**27**(3):221-34. doi:10.1007/s10822-013-9644-8
44. Friesner RA, Banks JL, Murphy RB*, et al.* Glide: a new approach for rapid, accurate docking and scoring. 1. Method and assessment of docking accuracy. *J Med Chem* 2004;**47**(7):1739-49. doi:10.1021/jm0306430
45. Halgren TA, Murphy RB, Friesner RA*, et al.* Glide: a new approach for rapid, accurate docking and scoring. 2. Enrichment factors in database screening. *J Med Chem* 2004;**47**(7):1750-9. doi:10.1021/jm030644s
46. Harder E, Damm W, Maple J*, et al.* OPLS3: A Force Field Providing Broad Coverage of Drug-like Small Molecules and Proteins. *J Chem Theory Comput* 2016;**12**(1):281-96. doi:10.1021/acs.jctc.5b00864
47. Liu S, Song X, Chrunyk BA*, et al.* Crystal structures of interleukin 17A and its complex with IL-17 receptor A. *Nat Commun* 2013;**4**:1888. doi:10.1038/ncomms2880
